# Supplementary material for: The human VGLUT3-pT8I mutation elicits uneven striatal DA signaling, food or drug maladaptive consumption in male mice
Source: Nat Commun. 2024 Jul 7;15:5691. doi: 10.1038/s41467-024-49371-1 (PMC11227582; doi:10.1038/s41467-024-49371-1)
Supplement: Supplementary file 1 — Supplementary information [file 41467_2024_49371_MOESM1_ESM.docx]

**Supplementary information**

**Title: The human VGLUT3-pT8I mutation elicits uneven striatal DA signaling, food or drug maladaptive consumption in male mice.**

Mathieu Favier, Elena Martin Garcia, Romain Icick, Camille de Almeida, Joachim Jehl, Mazarine Desplanque**,** Johannes Zimmermann, Annabelle Henrion, Nina Mansouri-Guilani, Coline Mounier, Svethna Ribeiro, Fiona Henderson, Andrea Geoffroy, Sebastien Mella, Odile Poirel, Véronique Bernard, Véronique Fabre, Yulong Li, Christian Rosenmund, Stéphane Jamain, Florence Vorspan, Alexandre Mourot, Philibert Duriez, Leora Pinhas, Rafael Maldonado, Nicolas Pietrancosta, Stéphanie Daumas and Salah El Mestikawy

**This file includes:**

- Statistical data Tables S1-S5 for Figures 1-5
- Supplementary Material and Methods
- Supplementary Tables S6-9 for Figure 1
- Supplementary Figures S1-6
- Statistical data Tables S10-13 for Supplementary Figures S1-4
- Supplementary References

**Supplementary Material and Methods**

**Table S1: Statistics for Fig. 1 and Supplementary Fig. S1**

| Bivariate associations between VGLUT1, VGLUT2 and VGLUT3 gene variants and phenotypic variables in patients with addictive disorders | | | |
| --- | --- | --- | --- |
| Kruskal-Wallis test of SAPS total score between patients with p.T8I variant (n=3), other VGLUT3 variants (n=3) and no VGLUT3 variant (n=4) | | | |
| T8I vs. other SLC17A8 vs. no SLC17A8 mutation | | | |
| SAPS scores, median (IQR):  T8I, 10 (10-11)  Other SLC17A8 variants, 3 (2.3-3.5)  No SLC17A8 variants, 5 (3-9) | | Kruskal-Wallis chi-squared=8.16, df=2,  p-value=0.0169  Effect size=0.0147 (small) | |
| *Without imputation of SAPS scores (Fig. S1)* | | | |
| SAPS scores, median (IQR):  T8I, 1& (10.5-11)  Other SLC17A8 variants, 3 (2.3-3.5)  No SLC17A8 variants, 6 (3-9) | | Kruskal-Wallis chi-squared=6.88, df=2,  p-value=0.032  Effect size= 0.0094 (small) | |
| Missense SLC17A6 or SLC17A7 vs. no mutation | | | |
| SAPS scores [median (IQR)]:  SLC17A6 or SLC17A7, 5 (3-7.5)  No mutation, 5 (3-9) | | Wilcoxon rank sum test with continuity correction, W = 517, p-value = 0.9197 | |
| Fisher exact test for the presence or absence of comorbid lifetime substance use disorders as a function of the presence of SLC17A8 variants | | | |
| Cocaine | Disorder present: 5 (63%) T8I, 5 (71%) other variants  Disorder absent: 3 (38%) T8I, 2 (29%) other variants | | p-value=0.561 |
| Alcohol | Disorder present: 2 (40%) T8I, 1 (17%) other variant  Disorder absent: 3 (60%) T8I, 5 (83%) other variants | | p-value=0.03 |
| Opioids | Disorder present: 8 (100%) T8I, 6 (86%) other variants  Disorder absent: 0 T8I, 1 (14%) other variant | | p-value=0.288 |
| Cannabis | Disorder present: 4 (80%) T8I, 2 (67%) other variants  Disorder absent: 1 (20%) T8I, 1 (33%) other variant | | p-value=0.349 |

**Table S2: Statistics for Fig. 2**

| Immunoautoradiography (Fig. 2h) | | |
| --- | --- | --- |
| Unpaired t-test - WT mice *vs* VGLUT3^T8i/T8i^ mice | | |
| Brain area | n | p-value |
| Cx | 7-8 | 0.996 |
| St | 7-8 | 0.541 |
| NAc | 7-8 | 0.531 |
| DSt | 7-8 | 0.161 |
| DMS | 7-8 | 0.222 |
| DLS | 7-8 | 0.131 |
| Hi | 6-8 | 0.284 |
| DG | 6-8 | 0.149 |
| CA1 | 6-8 | 0.205 |
| CA3 | 6-8 | 0.604 |
| DR | 7-8 | 0.494 |
| MnR | 7-8 | 0.276 |

| STED microscopy - relative frequency distribution (Fig. 2p) | |
| --- | --- |
| Kolmogorov-Smirnov test - WT mice *vs* VGLUT3^T8i/T8i^ mice | |
| n | p-value |
| 199 | 0.218 |
| STED microscopy - % of NNDs (Fig.2q) | |
| Chi-squared test - NNDs < 95nm vs > 95 nm and VGLUT3^T8i/T8i^ mice *vs* WT mice | |
| n | p-value |
| 2-2 | 0.439 |

**Table S3: Statistics for Fig. 3**

| ATP-dependent [^3^H] Glutamate uptake (Fig. 3e) | | |
| --- | --- | --- |
|  | One-way ANOVA | |
|  | WT mice vs VGLUT3-KO mice vs VGLUT3^T8i/T8i^ mice | |
|  | F-value | p-value |
| Genotype | F_2,15_=2.15 | 0.001 |
|  | Tukey’s test post hoc analysis | |
|  | n | p-value |
| WT mice vs VGLUT3-KO mice | 6-6 | 0.005 |
| WT mice vs VGLUT3^T8i/T8i^ mice | 6-6 | 0.851 |
| VGLUT3-KO mice vs VGLUT3^T8i/T8i^ mice | 6-6 | 0.002 |

| mEPSC - amplitude (Fig. 3f) | | |
| --- | --- | --- |
|  | Kruskal-Wallis test | |
|  | WT mice *vs* VGLUT3^T8i/T8i^ mice *vs* VGLUT1-KO mice | |
|  | n | *p*-value |
| Genotype | 48-39-49 | <0.001 |
|  | Dunn’s test post hoc analysis | |
|  | n | *p*-value |
| WT mice *vs* VGLUT3^T8i/T8i^ mice | 48-39 | >0.999 |
| VGLUT3^T8i/T8i^ mice *vs* VGLUT1-KO mice | 39-49 | <0.001 |
| WT mice *vs* VGLUT1-KO mice | 48-49 | <0.001 |

| mEPSC - frequency (Fig. 3g) | | |
| --- | --- | --- |
|  | Kruskal-Wallis test | |
|  | WT mice *vs* VGLUT3^T8i/T8i^ mice *vs* VGLUT1-KO mice | |
|  | n | *p*-value |
| Genotype | 48-39-49 | <0.001 |
|  | Dunn’s test post hoc analysis | |
|  | n | *p*-value |
| WT mice *vs* VGLUT3^T8i/T8i^ mice | 48-39 | >0.999 |
| VGLUT3^T8i/T8i^ mice *vs* VGLUT1-KO mice | 39-49 | <0.001 |
| WT mice *vs* VGLUT1-KO mice | 48-49 | <0.001 |

| mEPSC - charge (Fig. 3h) | | |
| --- | --- | --- |
|  | Kruskal-Wallis test | |
|  | WT mice *vs* VGLUT3^T8i/T8i^ mice *vs* VGLUT1-KO mice | |
|  | n | *p*-value |
| Genotype | 48-39-49 | <0.001 |
|  | Dunn’s test post hoc analysis | |
|  | n | *p*-value |
| WT mice *vs* VGLUT3^T8i/T8i^ mice | 48-39 | >0.999 |
| VGLUT3^T8i/T8i^ mice *vs* VGLUT1-KO mice | 39-49 | <0.001 |
| WT mice *vs* VGLUT1-KO mice | 48-49 | <0.001 |

| Vesamicol sensitive [^3^H] ACh uptake (Fig. 3i) | | |
| --- | --- | --- |
|  | Two-way ANOVA | |
|  | WT mice *vs* VGLUT3^T8i/T8i^ mice | |
|  | F-value | p-value |
| Genotype | F_1,26_=12.98 | 0.001 |
| Treatment (glutamate) | F_1,26_=38.82 | <0.001 |
| Genotype X Treatment (glutamate) | F_1,26_=3.921 | 0.058 |
|  | Bonferroni’s test post hoc analysis | |
|  | n | p-value |
| WT mice / Glu- *vs* VGLUT3^T8i/T8i^ mice / Glu- | 8-7 | >0.999 |
| WT mice / Glu- *vs* WT mice / Glu+ | 8-8 | <0.001 |
| WT mice / Glu- *vs* VGLUT3^T8i/T8i^ mice / Glu+ | 8-7 | 0.447 |
| VGLUT3^T8i/T8i^ mice / Glu- *vs* WT mice / Glu+ | 7-8 | <0.001 |
| VGLUT3^T8i/T8i^ mice / Glu- *vs* VGLUT3^T8i/T8i^ mice / Glu+ | 7-7 | 0.044 |
| WT mice / Glu+ *vs* VGLUT3^T8i/T8i^ mice / Glu+ | 8-7 | 0.003 |

| ACh fiber photometry – mean peak amplitude (Fig. 3l) | |
| --- | --- |
| Wilcoxon test - WT mice *vs* VGLUT3^T8i/T8i^ mice | |
| n | p-value |
| 7-7 | 0.382 |

| ACh fiber photometry – frequency of events (Fig. 3m) | |
| --- | --- |
| Unpaired t-test - WT mice *vs* VGLUT3^T8i/T8i^ mice | |
| n | p-value |
| 7-7 | 0.009 |
| Wilcoxon rank sum test with continuity correction (non-paired) | |
| 7-7 | 0.017  (W 43.5) |

| ACh fiber photometry – cumulative distribution of inter-event interval (Fig. 3n) | |
| --- | --- |
| Kolmogorov-Smirnov test - WT mice *vs* VGLUT3^T8i/T8i^ mice | |
| n | p-value |
| 55-22 | 0.001 |

| Voltammetry - DA release - dorsomedial striatum (DMS) (Fig. 3o) | | |
| --- | --- | --- |
|  | WT mice *vs* VGLUT3^T8i/T8i^ mice (n=17-14) | |
|  | Two-way ANOVA repeated measures | |
|  | F-value | p-value |
| Genotype | F_1,29_=5.63 | 0.024 |
| Time | F_2.499, 72.46_=39.76 | <0.001 |
| Genotype x Time | F_399, 11571_=1.84 | <0.001 |

| Voltammetry – Max. DA release - dorsomedial striatum (DMS) (Fig. 3p) | |
| --- | --- |
| Unpaired t-test - WT mice *vs* VGLUT3^T8i/T8i^ mice | |
| n | p-value |
| 17-14 | 0.03 |

| Voltammetry - DA release - dorsolateral striatum (DLS) (Fig. 3q) | | |
| --- | --- | --- |
|  | WT mice *vs* VGLUT3^T8i/T8i^ mice (n=13-14) | |
|  | Two-way ANOVA repeated measures | |
|  | Controls *vs* mutant littermates | |
|  | F-value | p-value |
| Genotype | F_1,25_=0.049 | 0.827 |
| Injections | F_1.797,44.92_=21.1 | <0.001 |
| Genotype x Injections | F_399,9975_=1.008 | 0.448 |

| Voltammetry – Max. DA release - dorsolateral striatum (DLS) (Fig. 3r) | |
| --- | --- |
| Unpaired t-test - WT mice *vs* VGLUT3^T8i/T8i^ mice | |
| n | p-value |
| 13-14 | 0.849 |

**Table S4: Statistics for Fig. 4**

| Locomotor activity (Fig. 4a) | | |
| --- | --- | --- |
|  | WT mice *vs* VGLUT3^T8i/T8i^ mice (n=8-8) | |
|  | Two-way ANOVA repeated measures | |
|  | F-value | p-value |
| Genotype | F_1,14_=0.437 | 0.519 |
| Time | F_5.213,72.98_=13.14 | <0.001 |
| Genotype x Time | F_30,420_=0.794 | 0.776 |

| Anxiety (EPM) - number of entries (Fig. 4b) | |
| --- | --- |
| Unpaired t-test - WT mice *vs* VGLUT3^T8i/T8i^ mice (n=12-12) | |
|  | p-value |
| Closed arms | 0.926 |
| Open arms | 0.632 |

| Anxiety (EPM) - time in open arms (%) (Fig. 4C) | |
| --- | --- |
| Unpaired t-test - WT mice *vs* VGLUT3^T8i/T8i^ mice | |
| n | p-value |
| 12-12 | 0.815 |

| Anxiety (EPM) - transitions from C to O arms (Fig. 4d) | |
| --- | --- |
| Unpaired t-test - WT mice *vs* VGLUT3^T8i/T8i^ mice | |
| n | p-value |
| 12-12 | 0.832 |

| Anxiety - Marble burying test - number of marbles buried (Fig. 4e) | | |
| --- | --- | --- |
|  | WT mice *vs* VGLUT3^T8i/T8i^ mice (n=12-12) | |
|  | Two-way ANOVA repeated measures | |
|  | F-value | p-value |
| Genotype | F_1,22_=0.374 | 0.547 |
| Time | F_1.308,28.78_=81.24 | <0.001 |
| Genotype x Time | F_9,198_=0.632 | 0.769 |

| Cocaine self-administration - FR1 and FR3 Training (Fig. 4f)  (Active nosepokes) | | |
| --- | --- | --- |
|  | WT mice *vs* VGLUT3^T8i/T8i^ mice (n=14-11) | |
|  | Two-way ANOVA repeated measures | |
|  | F-value | p-value |
| Genotype | F_1,23_=1.641 | 0.2129 |
| Time | F_9,207_=8.792 | <0.001 |
| Genotype x Time | F_9,207_=0.972 | 0.4641 |

| Cocaine self-administration - Acquisition (Fig. 4g) | |
| --- | --- |
| Chi-squared test - WT mice *vs* VGLUT3^T8i/T8i^ mice | |
| n | p-value |
| 14-11 | 0.010 |

| Cocaine self-administration - Breaking point (Fig. 4h) | |
| --- | --- |
| Unpaired t-test - WT mice *vs* VGLUT3^T8i/T8i^ mice | |
| n | p-value |
| 14-11 | 0.60 |

| Cocaine self-administration - Extinction (Fig. 4i) | | |
| --- | --- | --- |
|  | WT mice *vs* VGLUT3^T8i/T8i^ mice (n=14-11) | |
|  | Two-way ANOVA repeated measures | |
|  | F-value | p-value |
| Genotype | F_1,23_=0.1029 | 0.7513 |
| Time | F_21,483_=9.025 | <0.001 |
| Genotype x Time | F_21,483_=0.7128 | 0.8215 |

| Cocaine self-administration - Cue-induced reinstatement (Fig. 4j) | |
| --- | --- |
| Unpaired t-test - WT mice *vs* VGLUT3^T8i/T8i^ mice | |
| n | p-value |
| 14-11 | 0.041 |

| Cue induced reinstatement (Fig. 4k) | | |
| --- | --- | --- |
|  | WT mice *vs* VGLUT3^T8i/T8i^ mice (n=14-11) | |
| Acquisition vs extinction in WT | Paired t-test | <0.01 |
| Acquisition vs extinction in VGLUT3^T8i/T8i^ mice | Paired t-test | <0.05 |
| Extinction vs cue-indiced reinstatement in WT | Paired t-test | <0.01 |
| Acquisition vs cue-indiced reinstatement in VGLUT3^T8i/T8i^ mice | Paired t-test | <0.01 |
| WT mice *vs* VGLUT3^T8i/T8i^ mice in acquisition | Unpaired t-test | 0.67 |
| WT mice *vs* VGLUT3^T8i/T8i^ mice in extinction | Unpaired t-test | 0.80 |
| WT mice *vs* VGLUT3^T8i/T8i^ mice in cue induced reinstatement | Unpaired t-test | <0.05 |

**Table S5: Statistics for Fig. 5**

| Sucrose self-administration – FR1 training (Fig. 5a) | | |
| --- | --- | --- |
|  | WT mice *vs* VGLUT3^T8i/T8i^ mice (n=12-12) | |
|  | Two-way ANOVA repeated measures | |
|  | F-value | p-value |
| Genotype | F_1,22_=0.0001 | 0.992 |
| Time | F_3.064,67.41_=54.11 | <0.001 |
| Genotype x Time | F_15,330_=0.548 | 0.912 |

| Devaluation test (Fig. 5b) | | |
| --- | --- | --- |
|  | WT mice *vs* VGLUT3^T8i/T8i^ mice (n=11-9) | |
|  | Two-way ANOVA repeated measures | |
|  | F-value | p-value |
| Genotype | F_1,36_=1.0001 | 0.6103 |
| Value | F_1,36_=54.11 | 0.0131 |
| Genotype x Value | F_1,36_=0.548 | 0.0102 |

| Devaluation test (Fig. 5b) | |
| --- | --- |
| Paired t-test - WT mice/valued *vs* WT mice/devalued | |
| n | p-value |
| 11-9 | 0.001 |
| Paired t-test - VGLUT3^T8i/T8i^ mice/valued *vs* VGLUT3^T8i/T8i^ mice/devalued | |
| n | p-value |
| 11-9 | 0.945 |

| Food addiction - FR1 and FR5 training (Fig. 5c) | | |
| --- | --- | --- |
|  | WT mice *vs* VGLUT3^T8i/T8i^ mice (n=14-13) | |
|  | Mixed effect model (REML) | |
|  | F-value | p-value |
| Genotype | F_1,25_=0.059 | 0.811 |
| Time | F_9.073,223.9_=5.468 | <0.001 |
| Genotype x Time | F_123,3036_=1.43 | 0.002 |

| Food addiction - Persistence (Fig. 5d) | |
| --- | --- |
| Unpaired t-test - WT mice *vs* VGLUT3^T8i/T8i^ mice | |
| n | p-value |
| 14-13 | 0.113 |
| Mann-Whitney - WT mice *vs* VGLUT3^T8i/T8i^ mice | |
| 14-13 | 0.2088  (U=67) |

| Food addiction - Motivation (Fig. 5e) | |
| --- | --- |
| Unpaired t-test - WT mice *vs* VGLUT3^T8i/T8i^ mice | |
| n | p-value |
| 14-13 | 0.677 |

| Food addiction - Compulsivity (Fig. 5f) | |
| --- | --- |
| Unpaired t-test - WT mice *vs* VGLUT3^T8i/T8i^ mice | |
| n | p-value |
| 14-13 | 0.597 |

| Food addiction - Percentage of addicted mice (Fig. 5g,h) | |
| --- | --- |
| Chi-squared test - Addicted *vs* non addicted and VGLUT3^T8i/T8i^ mice *vs* WT mice | |
| n | p-value |
| 14-13 | 0.918 |

| Food addiction - Pearson’s correlations addiction-like criteria/persistence (Fig. 5i) | |
| --- | --- |
| WT mice | |
| r^2^ | p-value |
| 0.334 | 0.031 |
| VGLUT^T8I/T8i^ mice | |
| r^2^ | p-value |
| 0.2 | 0.126 |

| Food addiction - Pearson’s correlations addiction-like criteria/motivation (Fig. 5j) | |
| --- | --- |
| WT mice | |
| r^2^ | p-value |
| 0.295 | 0.045 |
| VGLUT^T8I/T8i^ mice | |
| r^2^ | p-value |
| 0.305 | 0.051 |

| Food addiction - Pearson’s correlations addiction-like criteria/compulsivity (Fig. 5k) | |
| --- | --- |
| WT mice | |
| r^2^ | p-value |
| 0.414 | 0.013 |
| VGLUT^T8I/T8i^ mice | |
| r^2^ | p-value |
| 0.405 | 0.011 |

| Sucrose binge-like overconsumption model - Sucrose intake H0-H4 (Fig. 5l) | | |
| --- | --- | --- |
|  | WT mice *vs* VGLUT3^T8i/T8i^ mice (n=10-10) | |
|  | Two-way ANOVA repeated measures | |
|  | F-value | p-value |
| Genotype | F_1,18_=0.96 | 0.34 |
| Time | F_15,270_=164.8 | <0.001 |
| Genotype x Time | F_15,270_=1.432 | 0.132 |

| Sucrose binge-like overconsumption model - Sucrose intake H0-H1 (Fig. 5m) | | |
| --- | --- | --- |
|  | WT mice *vs* VGLUT3^T8i/T8i^ mice (n=10-10) | |
|  | Two-way ANOVA repeated measures | |
|  | F-value | p-value |
| Genotype | F_1,18_=3.692 | 0.071 |
| Time | F_5.587,100.6_=119.5 | <0.001 |
| Genotype x Time | F_15,270_=3.066 | <0.001 |

| Sucrose binge-like overconsumption model - Sucrose intake H1-H4 (Fig. 5n) | | |
| --- | --- | --- |
|  | WT mice *vs* VGLUT3^T8i/T8i^ mice (n=10-10) | |
|  | Two-way ANOVA repeated measures | |
|  | F-value | p-value |
| Genotype | F_1,18_=0.027 | 0.872 |
| Time | F_15,270_=42.37 | <0.001 |
| Genotype x Time | F_15,270_=0.319 | 0.993 |

| Activity-based anorexia model - mice up to 75% of baseline of BW (Fig. 5o) | |
| --- | --- |
| WT mice *vs* VGLUT3^T8i/T8i^ mice (n=10-10) | |
| Kaplan-Meier test | |
| Log-rank (Mantel-Cox) post hoc comparison | p<0.001 |
| Gehan-Breslow-Wilcoxon post hoc comparison | p<0.001 |

| Activity-based anorexia model - mice up to 75% of baseline of BW (Fig. 5p) | |
| --- | --- |
| WT mice-saline *vs* WT mice-donepezil (n=10-10) | |
| Kaplan-Meier test | |
| Log-rank (Mantel-Cox) post hoc comparison | p=0.819 |
| Gehan-Breslow-Wilcoxon post hoc comparison | p=0.83 |

| Activity-based anorexia model - mice up to 75% of baseline of BW (Fig. 5q) | |
| --- | --- |
| VGLUT3^T8i/T8i^ mice-saline *vs* VGLUT3^T8i/T8i^ mice-donepezil (n=10-10) | |
| Kaplan-Meier test | |
| Log-rank (Mantel-Cox) post hoc comparison | p=0.006 |
| Gehan-Breslow-Wilcoxon post hoc comparison | p=0.004 |

**Supplementary Material and Methods**

**Eating disorders (EDs) sample**

1. Participants

EDs patients were recruited through the specialized Eating Disorder (ED) program from the Douglas Hospital for adults (McGill University, Montreal, Canada). This cohort included 269 consenting women with a [DSM-IV-TR](https://www-sciencedirect-com.proxy.insermbiblio.inist.fr/topics/medicine-and-dentistry/dsm-iv-tr) ([American Psychiatric Association, 2000](https://www-sciencedirect-com.proxy.insermbiblio.inist.fr/science/article/pii/S0165178110006517?via%3Dihub#bb0005)) diagnosis of anorexia nervosa (n=73) bulimia nervosa (n=116), eating disorders not otherwise specified (EDNOS, n= 80). Of these women, 116 (91%) could provide blood samples for genotyping.

Subjects were between the ages of 18 and 44 year-old; [Body Mass Index](https://www-sciencedirect-com.proxy.insermbiblio.inist.fr/topics/medicine-and-dentistry/body-mass-index) (BMI: kg/m^2^) fell between 18 and 34 for bulimic participants (mean = 21.39 ± 3.89). Within the eating-disordered group 35 (29%) had a secondary-level education, 47 (39%) college-level, and 38 (32%) university level.

The Quebec population from which this sample was drawn is skewed towards individuals of Caucasian, Western-European descent. Consequently, our bulimic sample included mainly Caucasians (*n* = 125, or 96.9% of the sample), with rare Blacks (*n* = 2, or 1.6% of the sample) and Asians (*n* = 2, or 1.6% of the sample). Data on ethnicity were available for only 43 (43.9%) of the normal-eater participants. However, these data indicated racial composition of our control group: 33 (76.7%) Caucasians, 5 (11.6%) Blacks and 5 (11.6%) Asians.

1. Measures

EDs diagnosis and symptoms were assessed using the Eating Disorders Examination (EDE^1^), a semi-structured interview assessing the presence and severity of core EDs symptoms. The current “Gold Standard” for EDs diagnosis, the EDE has established reliability and validity. Screening for comorbid (past 12 months) [DSM-IV](https://www-sciencedirect-com.proxy.insermbiblio.inist.fr/topics/medicine-and-dentistry/dsm-iv) Axis-I disorders was accomplished using the [Structured Clinical Interview for DSM-IV](https://www-sciencedirect-com.proxy.insermbiblio.inist.fr/topics/medicine-and-dentistry/structured-clinical-interview-for-dsm-disorders) Axis-I disorders (SCID-I: <https://www.ptsd.va.gov/professional/assessment/adult-int/scid-ptsd-module.asp>).

**Substance use disorders (SUDs) sample**

1. Participants

Treatment-seeking outpatients attending tertiary care programs in the Paris area were recruited through two multicenter research protocols. Participants were French-speaking, 18+ year-old individuals, seeking treatment in any of the participating centers. Further inclusion criteria were:

- protocol one (seven sites, 2008-2012) = receiving stable methadone treatment for three months or more for treating opioid use disorder ^2^;
- protocol two (nine sites, 2012-2016) = any past-year cocaine use ^3^.

For the present study, participants had to fulfil criteria for either lifetime opiate or cocaine abuse/dependence named substance use disorders according to DSM-IV-TR ^4^ (the DSM5 was not released at the time of the study). Patients were excluded if they were undergoing compulsory treatment or were unable to consent for any other reason (non-French speaking, major cognitive impairment).

Both protocols were approved by the local ethics committees (*CPP Ile-de-France* VI for study one and CPP Ile de France IV for study two) and preregistered (clinicaltrials.gov NCT00894452 and NCT01569347, respectively), and by the relevant Institutional Review Board for further analyses of the combined sample [CEEI from the *Institut de la Santé et de la Recherche Médicale* (INSERM), IRB00003888 in July 2015]. All participants provided written informed consent, and study records were continuously monitored by the local research administration (*Unité de Recherche Clinique*) to ensure their conformity to the original protocols. The authors assert that all procedures contributing to this work comply with the ethical standards of the relevant national and institutional committees on human experimentation and with the Helsinki Declaration of 1975, as revised in 2008.

1. Biological sampling and genotyping

*SLC17A8 sequence analysis in the ED cohort.*

For each individual, genomic DNA was isolated from blood leukocytes using a standard procedure. The SLC17A8 coding regions were amplified before sequencing using the BigDye Terminator v3.1 Cycle Sequencing Kit (Life Technologies, Carlsbad, CA, USA) and run on a 16-Capillary ABI PRISM 3130xl genetic analyzer (Life Technologies, Carlsbad, CA, USA). All primers used for the PCR amplifications and sequence analyses are available on request. Genotyping of 390 additional controls of African origin was performed to examine the allele frequency of rs45610843.

*Genome-wide quality control and SLC17A8 genotyping in the SUD cohort*

PLINK was used for quality control, following on a consensus procedure ^5^ (see flowchart, Supplementary Figure 1), at the whole-sample (N=576) and genome-wide (566,932 SNPs) levels, excluding individuals/markers showing: sex discrepancy, >2% genotype missingness (per marker and per individual), deviation from Hardy-Weinberg Equilibrium at *p* <10^-6^, cryptic relatedness (Identity-by-Descent pi-hat threshold of 0.2, *i.e.* 2^nd^ degree relatedness), leaving 525,681 variants and 524 individuals with a total genotyping rate =0.999409. Genotypes were then imputed using the Michigan state University server (<https://imputationserver.sph.umich.edu/index.html#!pages/home>), which comprises quality check, phasing (Eagle 4.2) and imputation (minimac V4), yielding 7,527,426 markers at the genome-wide level after post-imputation QC. The DNA array eventually comprised 321 allelic markers on *SLC17A8* (VGLUT3 gene) - see Supplementary Figure 1and Supplementary Table 3).

1. Additional consideration upon SAPS

Given that 25 SAPS scores were missing and given our focus on the rare variant p.T8I) we decided to impute SAPS scores, by subscale, based on the mode of each genotype group. The imputed value for SAPS-delusion subscale was the most frequent value of the SAPS-delusion subscale in a given genotype group. This yielded 363 analyzable data compared to 338 initially without imputation. Here, we show the same graph (Figure 1c) without imputation (N =338) showing the significant difference in SAPS total score across the three groups and between T8I carriers and patients without mutation (Supplementary Figure 1). Thus, imputation allowed us to maximize our sample size, while eliciting similar findings.

The SAPS has previously been used as a useful proxy of the severity of CUD. It appears to vary according to both the heaviness of cocaine use and the propensity of individuals to develop psychotic and motor symptoms. Comprehensive methods assessing the severity of substance use disorders in general have been used ^6^. However, these methods are lengthy and could have compromised recruitment rate in the study.

1. Bayesian analyses

Due to the very small size of the genotypic groups for *SLC17A8*, we performed Bayesian non-parametric analyses implemented in JASP software version 0.17.1. We set a relatively conservative Cauchy prior at 0.47 (corresponding to an hypothetical effect size =-1.2 / 1.2), and show the resulting Bayes factor_01_, which yields the probability that genetically-informed cocaine-related variables distributions significantly differ from prior distributions (“theoretical”, *i.e.* not under genotypic influence). The Bayes factor_01_ can be further interpretated as strength of evidence toward/against the difference, according to thresholds that have been summarized here: <https://wires.onlinelibrary.wiley.com/doi/10.1002/wics.1523>. Of note, these analyses can only be performed pairwise, so that we compared T8I carriers to other *SLC17A8* and to non-mutation carriers separately.

**gnomAD reference sample**

We examined the genotypes for rs45610843 in the gnomAD reference panel v4.0.0, which comprises 806,886 individuals genotyped by whole-genome array or sequencing (<https://gnomad.broadinstitute.org>). We chose this sample over other references (*e.g.* 1000 genomes) to increase representativeness due to sample size. There where 1670 rs45610843 carriers (including four homozygotes) - totalizing 1674 effect alleles, leaving 1,612,098 non-effect alleles. There were nine heterozygous rs45610843 carriers in our total (ED+SUD, N =793) clinical sample, thus nine effect alleles, leaving 784 * 2 +9=1595 non-effect alleles. These allelic counts were used to perform Fisher exact tests to compare allelic frequencies between the reference population and the SUD samples. We had to mix all ancestries given the mixed EUR and AFR genetically-verified ancestry of rs45610843 carriers within our clinical sample (data not shown, available upon request to authors). It is noticeable, however, that the SUD and the gnomAD reference panel had significantly different population admixture, probably due to differences in Asian and North-African subjects (most of them in “other” in the SUD sample and not mentioned specifically in gnomAD) (Fisher exact test using the numbers provided at https://gnomad.broadinstitute.org/stats, *p* =0.0004998).

**Operant model of reinstatement maintained by cocaine self-administration**

*Surgery and drugs*

Mice were anesthetized by a mixture of ketamine hydrochloride (Imalgène; Merial Laboratorios S.A., Barcelona, Spain; 75 mg/kg) and medetomidine hydrochloride (Domtor; Esteve, Barcelona, Spain; 1 mg/kg) dissolved in sterile 0.9% physiological saline. This anesthetic mixture was administered intraperitoneally in an injection volume of 10 ml/kg of body weight. After surgery, a subcutaneous injection of atipamezole hydrochloride (Revertor; Virbac, Barcelona, Spain; 2.5 mg/kg of body weight) was administered to reverse the anesthesia. Additionally, mice received a subcutaneous injection of meloxicam (Metacam; Boehringer Ingelheim, Rhein, Germany; 2 mg/kg of body weight) and an intraperitoneal injection of gentamicin (Genta-Gobens; Laboratorios Normon, S.A., Madrid, Spain; 1 mg/kg of body weight) all previously dissolved in sterile 0.9% physiological saline. For operant conditioning maintained by cocaine, cocaine hydrochloride (Sigma-Aldrich, Saint Louis, MO, USA) was dissolved in a saline solution (0.9% NaCl w/v). The self-administration intravenous infusion parameters were 0.5 mg/kg/infusion of 23µl. The catheter's patency was evaluated by thiopental sodium (5 mg/ml) (Braun Medical S.A, Barcelona, Spain) dissolved in distilled water and delivered by infusion of 0.1 ml through the intravenous catheter.

During surgery, mice were placed on a heating pad (30°C). A catheter was inserted in the right jugular vein until the tip reached the right atrium level; it was attached to the vein and held in place by a dab of silicone on the tubing. The distal end was passed subcutaneously over the shoulder for attachment in the mid-scapular region. After surgery, the mice were placed in the home cage on a heating pad (27°C) until they woke from anesthesia. To prevent infection, mice received daily i.p. injections of a 0.05 ml gentamicin solution (11.4 mg/ml, Gentalline®, Schering-Plough, Levallois- Perret, France) for 3 days. After each self-administration session, the catheters were flushed with a saline solution containing heparin (30 IU/ml), which tested their patency, especially when behavior appeared to deviate (±20%) from the previous session. The success rate for maintaining the patency of the catheter until the end of the cocaine self-administration training was 85 %. Thus, mice that did not show patency of the catheter were removed from the experiment.

*Cocaine self-administration apparatus*

Cocaine self-administration training was performed in operant chambers (Model ENV-307A-CT, Med Associates, Inc., Georgia, VT, USA) equipped with two holes, one randomly selected as the active hole and the other as the inactive. Pump noise and a stimulus-light located above active hole were paired with the delivery of the infusion. Chambers had grid floors and were housed in sound- and light-attenuated boxes equipped with fans to provide ventilation and ambient noise. When mice responded on the reinforced hole, the stimulus light went on, and a cocaine (0.5 mg/kg) infusion was delivered via a syringe mounted on a microinfusion pump (PHM-100A, Med Associates, Inc., Georgia, VT, USA) connected via Tygon tubing (0.96 mm o.d., Portex Fine Bore Polythene Tubing, Portex Limited, Hythe, Kent, UK) to a single channel liquid swivel (375/25, Instech Laboratories, Plymouth Meeting, PA, USA), and to the mouse intravenous (i.v.) catheter.

*Experimental design of the model of reinstatement of operant conditioning maintained by cocaine*

Animals were trained to acquire operant responding maintained by cocaine (0.5 mg/kg/infusion of 23µl, i.v.) under a fixed ratio 1 (FR1) (5 consecutive days) and FR3 (5 consecutive days). The criteria for acquisition of operant responding were achieved when mice maintained a stable response with less than 20% deviation from the mean of the total number of infusions earned in three consecutive sessions, with at least 75% responding on the reinforced nose-poke, and a minimum of 10 reinforcements per session^7, 8^. After the 10 FR sessions, animals were tested in a progressive ratio (PR) schedule where the response requirement to earn infusions escalated according to the following series: 1–2–3–5–12–18–27–40–60–90–135–200–300–450–675–1000. The maximum duration of the PR session was 4 h or until mice did not respond on any hole within 1 h, and it was performed only once. After the PR session, the thiopental test was applied, and only mice that showed patency of the catheter were moved to the extinction and relapse phases. The first extinction session occurred 48 h after the thiopental tests to avoid any possible influence of thiopental residual effects. During the extinction phase, the experimental conditions were similar to the acquisition sessions except that cocaine was not available and the cue light was not presented after active responding. Mice were given 2 h daily extinction sessions for 15 consecutive days until the criteria for extinction was achieved. The criteria were reached when during three consecutive sessions, mice responded on the active lever less than 30% of the responses reached in the three last acquisition days and made less than 15 active responses per session. After extinction, mice were tested for reinstatement. Cues-induced reinstatement was conducted under the same conditions used in the acquisition phase, except cocaine was not delivered. Each response on the active manipulandum in this phase led to the presentation of the cue light for 2 s. The reinstatement criterion was achieved when responding in the nose-poke doubled with respect to extinction


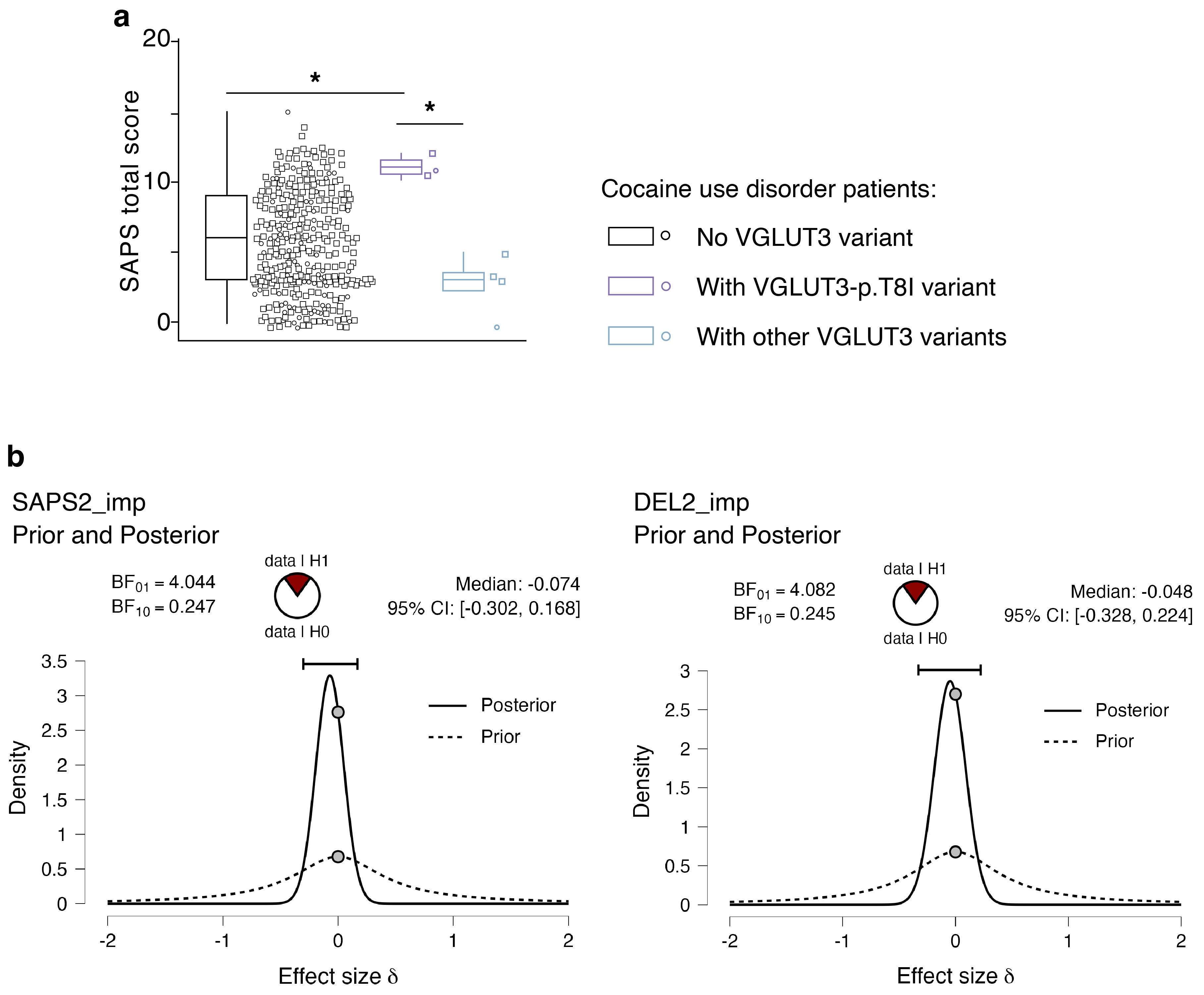


**Supplementary Figure 1**

(**a**) Boxplots with the same groups as in Figure 1C, without imputing SAPS total score (N =338).

Distribution of total SAPS score as a function of VGLUT3 variants (no variant vs. T8I vs. others) in the same patients with established cocaine use disorder as in Figure 1 with no imputation*.* N =338 biologically independent samples. Total SAPS score significantly differed across the three genotypes groups (Kruskal-Wallis p=0.032). Carriers of the p.T8I isoform had significantly higher SAPS score compared to patients without VGLUT3 mutation (two-sided Wilcoxon test p=0.04623), but not compared to other VGLUT3 mutations (two-sided Wilcoxon test, p=0.1).

(**b**) Bayes analysis of significant difference evidenced in Figure 1d.

Distribution of effect sizes after Bayesian Wilcoxon tests performed in JASP software for SAPS total score (SAPS2_imp, left pane) and delusion score (DEL2_imp, right panel). Median differences are those between carriers *vs.* non-carriers of VGLUT1&2 rare mutations. Circles display the respective probability of H1 (the fact that carriers have higher scores than non-carriers) and H0 (opposite hypothesis), indicating substantial support toward H0 [four times more likely according to Bayes Factor (BF_01_)]. The density curves describe the prior and posterior probability, showing that our pre-defined prior (Cauchy prior at 0.47) was at least moderately informative. Source data are provided as a Source Data file.

|  | Median (interquartile range) or N (%) | N |
| --- | --- | --- |
| Mean age (SD) | 26 (7) | 269 |
| Ancestry (declared): |  | 269 |
| Mixed/undetermined | 82 (30%) |  |
| Aboriginal | 3 (1%) |  |
| Asian | 2 (1%) |  |
| Black or African American | 5 (2%) |  |
| Caucasian | 175 (65%) |  |
| Mixed Native American and black or African American | 1 (0%) |  |
| Native Hawaiian or Other Pacific Islander | 1 (0%) |  |
| Diagnosis: |  | 269 |
| Anorexia Nervosa | 73 (27%) |  |
| Bulimia Nervosa | 116 (43%) |  |
| EDNOS | 80 (30%) |  |
| Mean age at onset of eating disorder (SD) | 16 (5) | 210 |
| Mean body mass index (SD) | 22 (4) | 210 |

**Table S6 Summary descriptive of the EDs sample.** EDNOS, eating disorder - not otherwise specified. All subjects from the ED sample were women.

|  | Median (interquartile range)  or N (%) | N |
| --- | --- | --- |
|  |  |  |
| Age | 40 (32-45) | 524 |
| Gender  (Women *vs.* Men) | 116 (22%) | 524 |
| Ancestry (genetically-informed)  European  Mixed American  African  East-Asian  South-Asian  Other (mostly between European and African) | 351 (67%)  20 (4%)  51 (10%)  1 (0%)  0 (0%)  101 (19%) | 524 |
| Cocaine use disorder | 378 (72%) | 524 |
| Age at onset of cocaine use disorder | 26 (20-32) | 341 |
| SAPS-CIP total | 7 (4-10) | 363 |
| SAPS-CIP, delusions | 2 (0-3) | 363 |
| SAPS-CIP, hallucinations | 1 (0-3) | 363 |
| SAPS-CIP, behavior | 3 (2-3) | 363 |
| SAPS-CIP, physical symptoms of *craving* | 1 (0-2) | 363 |
| Alcohol use disorder | 295 (56%) | 524 |
| Benzodiazepine use disorder | 200 (38%) | 524 |
| Cannabis use disorder | 316 (60%) | 524 |
| Opioid use disorder | 334 (64%) | 524 |

**Table S7 Summary descriptive of the SUDs sample with high-quality genotyping.** SAPS-CIP, scale for assessment of psychotic symptoms - cocaine-induced psychosis. Substance use disorder diagnoses are made on a lifetime basis.

| POS | Name | RsID | ref | alt | SIFT | POLYPHEN |
| --- | --- | --- | --- | --- | --- | --- |
| 100751192 | T8I | rs45610843 | C | T | **Deleterious - Low Confidence** | Benign |
| 100774588 | G71S | rs769738794 | G | A | Tolerated | Benign |
| 100774687 | V104I | rs373954823 | G | A | Tolerated | **Possibly damaging** |
| 100784813 | G130D | rs771270860 | G | A | **Deleterious** | **Probably damaging** |
| 100795633 | Y252S | rs765964381 | A | C | **Deleterious** | **Probably damaging** |
| 100797844 | M361T | rs543573169 | T | C | **Deleterious** | Benign |
| 100811837 | P443L | rs1218826366 | C | T | **Deleterious** | **Probably damaging** |

*N°7: mutation of the patient « PSC147 ». Not characterized in Sakae et al. 2015*

**Table S8 List of rare exonic allelic mutations analyzed in the study (8 p.T8I and 7 others).** Name and annotations of the 19 variants tested in the SUDs sample. Positions in base pairs refer to the hg19 version of the human genome. See supplementary Tables 2 & 3. P.T8I was identified by genotyping, other mutations by sequencing. Three rare *SLC17A8* mutations were available on the DNA array but were not detected in the clinical sample. POS, position on chromosome 12 (base pairs); ref, reference allele; alt, risk allele; minor allele frequencies in the 1000 genomes population =Africans (AFR), Mixed Americans (AMR), South-East Asians (EAS), Europeans (EUR), South Asians (SAS); consequence according to ENSEMBL v.102 annotation; SIFT, Sorting Intolerant from Tolerant ; PolyPhen, Polymorphism Phenotyping ; CADD, Combined Annotation Dependent Depletion.

| **Illumina Name** | **RsID** | **POS** | **ref** | **alt** | **AFR** | **AMR** | **EAS** | **EUR** | **SAS** | **Consequence** | **SIFT** | **PolyPhen** | **CADD** |
| --- | --- | --- | --- | --- | --- | --- | --- | --- | --- | --- | --- | --- | --- |
| exm1029287 | rs45610843 | 100751192 | C | T | 0.018 | 0.003 | None | None | None | coding nonsyn | Deleterious - Low Confidence | Benign |  |
| variant-26631 | rs371908574 | 100751200 | G | T |  |  |  |  |  | coding nonsyn:stop-gain |  |  |  |
| variant-26632 | rs369918682 | 100751275 | A | C |  |  |  |  |  | intronic |  |  | CADD=A/C:2.177422:21.100 |
| rs10778050 | rs10778050* | 100768827 | G | A | 0.57 | 0.68 | 0.869 | 0.442 | 0.718 | intronic |  |  | CADD=G/A:0.072831:3.812 |
| exm1749715 | rs141811441 | 100774609 | A | G | None | None | 0.004 | None | None | coding nonsyn | Tolerated | Benign |  |
| exm2271863 | rs7311096* | 100775956 | T | C | 0.977 | 0.7536 | 0.997 | 0.492 | 0.734 | intronic |  |  | CADD=T/C:-0.328393:0.300 |
| rs17030132 | rs17030132 | 100782614 | G | A | 0.114 | 0.182 | 0.133 | 0.083 | 0.156 | intronic |  |  | CADD=G/A:-0.059436:1.824 |
| var_12_100784785 | rs868172452 | 100784785 | T | C |  |  |  |  |  | intronic (splice_site) |  |  | CADD=T/C:4.489760:33.000 |
| exm1029315 | rs201585182 | 100784899 | G | A | None | None | None | 0.002 | None | coding nonsyn | Tolerated | Possibly Damaging |  |
| exm1029319 | rs142102133 | 100787205 | G | A |  |  |  |  |  | coding nonsyn | Deleterious | Possibly Damaging |  |
| exm1029320 | rs150737570 | 100787220 | C | T |  |  |  |  |  | coding nonsyn:stop-gain |  |  |  |
| variant-26636 | rs768546214 | 100790171 | G | - |  |  |  |  |  | non-coding, coding frameshift:stop-gain |  |  |  |
| indel-26638 | rs776808252 | 100797818 | G | A,T | None | None | 0.009 | 0.001 | 0.001 | non-coding, coding nonsyn\|nonsyn |  |  |  |
| exm1029353 | rs138307707* | 100797882 | C | T | 0.402 | 0.501 | 0.398 | 0.562 | 0.587 | intronic,3' downstream |  |  | CADD=C/T:-0.137011:1.091 |
| rs7965082 | rs7965082* | 100800193 | C | T | 0.253 | 0.434 | 0.16 | 0.5 | 0.393 | intronic, non-coding intronic |  |  | CADD=C/T:-0.056434:1.859 |
| rs11110370 | rs11110370* | 100804733 | T | G |  |  |  |  |  | non-coding, coding nonsyn:stop-gain |  |  |  |
| variant-26640 | rs781594104 | 100813750 | A | C |  |  |  |  |  | coding nonsyn | Tolerated | Benign or Probably damaging |  |
| exm1029377 | rs201679317 | 100813812 | G | A |  |  |  |  |  | coding nonsyn | Deleterious | Probably Damaging |  |
| indel-26641 | rs779092093 | 100813873 | C | - |  |  |  |  |  | 3' downstream, coding frameshift:stop-gain |  |  |  |

**Table S9 Name and annotations of the 19 *SLC17A8* variants available on the DNA array for the SUD sample**. Positions in base pairs refer to the hg19 version of the human genome. Variants marked with * are the frequent mutations tested in the specific analysis (2b in the study flowchart, see supplementary Figure 1**)**. RsID, variant name according to dbSNP version 150; POS, position on chromosome 12 (base pairs); ref, reference allele; alt, risk allele; minor allele frequencies in the 1000 genomes population =Africans (AFR), Mixed Americans (AMR), South-East Asians (EAS), Europeans (EUR), South Asians (SAS); consequence according to ENSEMBL v.102 annotation; SIFT, Sorting Intolerant from Tolerant ; PolyPhen, Polymorphism Phenotyping ; CADD, Combined Annotation Dependent Depletion

**
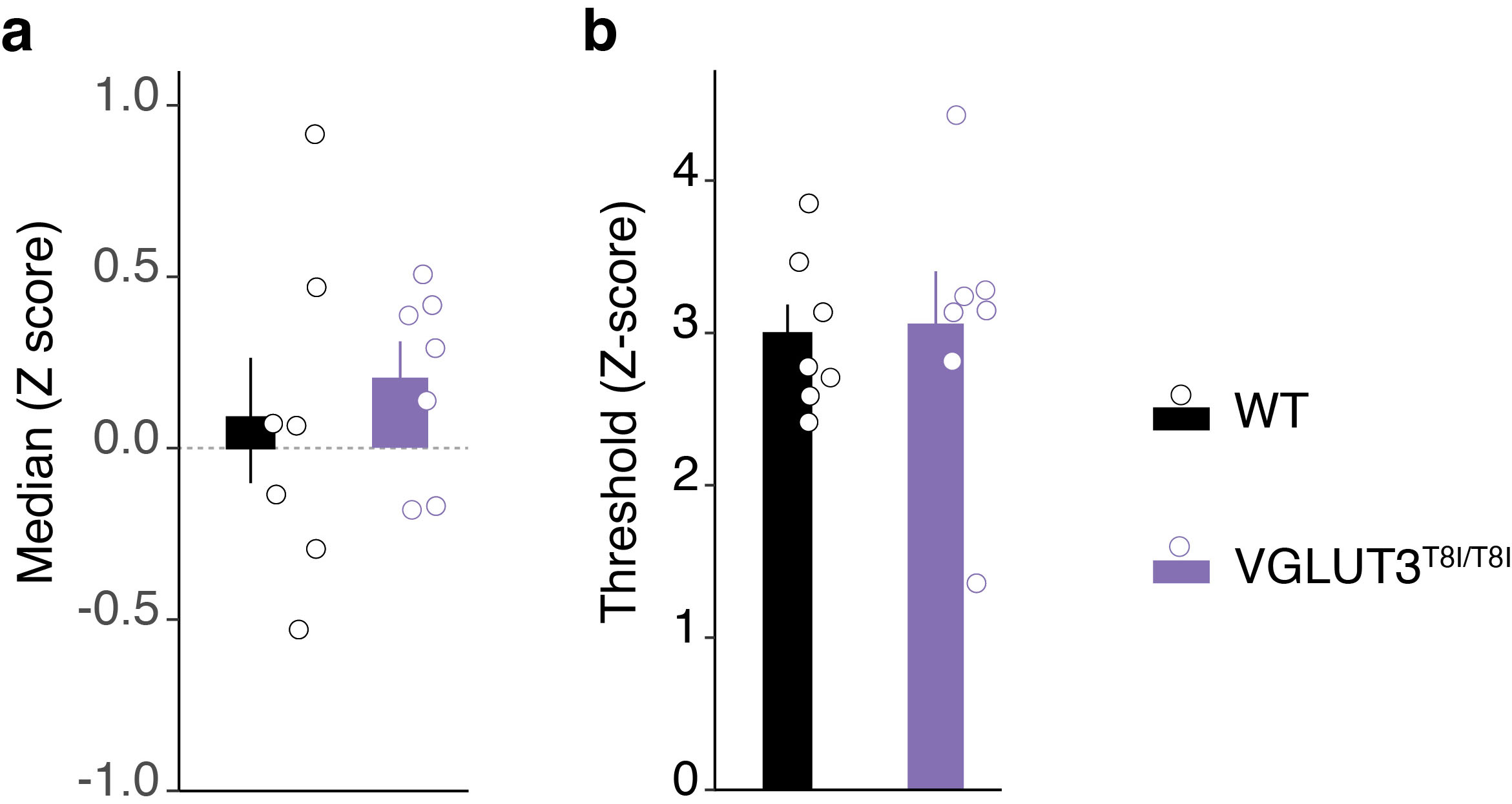
**

**Supplementary Fig. 2 In vivo fiber photometry of ACh release. a,** Median of the filtered trace for WT mice or VGLUT3^T8I/T8I^ mice (Wilcoxon rank sum test (non-paired) W=19, *p*= 0.535). **b**, Threshold for peak detection (local maxima over 3 MADs above the median) (Wilcoxon rank sum test (non-paired) W=19, *p*=0.535). Source data are provided as a Source Data file.


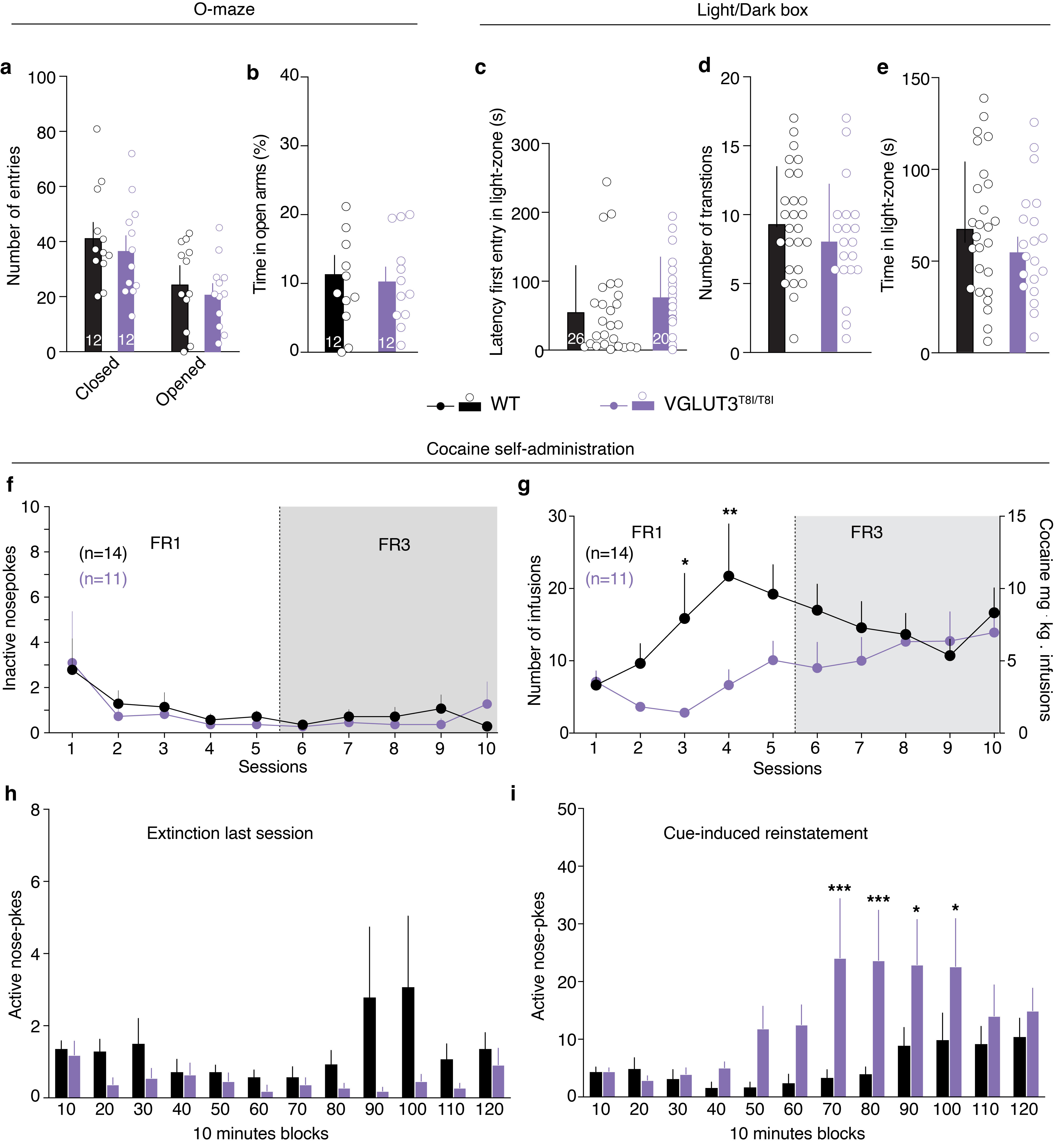


**Supplementary Fig. 3. Additional anxiety and cocaine self-administration data.** **a**, O-maze number of entries in closed or open arms (two-tailed unpaired t-test, NS). **b**, O-maze time spent in open arms versus closed arm genotype x time, F21,483=0.7128, p=0.8215, Two-way ANOVA repeated measures s (%) by WT mice or VGLUT3^T8I/T8I^ mice (two-tailed unpaired t-test, all NS). **c**, Light/dark box latency to enter the light compartment (two-tailed unpaired t-test, NS). **d**, Light/dark box number of transitions between the light and the dark compartment (two-tailed unpaired t-test, NS). **e**, Total time spent in the light compartment (two-tailed unpaired t-test, NS). **f**, Cocaine self-administration, number of inactive nosepokes during the acquisition (fixed ratio 1 (FR1) and 3 (FR3)) of self-administration (two-way ANOVA repeated measures NS). **g**, Cocaine self-administration, number of infusions (0.5mg/kg/infusion, left Y axis) and intake of cocaine in mg/kg/infusion of 23 µl (right axis) during acquisition of cocaine self-administration (genotype x time, F9,207=2.085, *p*<0.05, two-way ANOVA repeated measures, and LSD post hoc test days 3 **p*<0.05 and days 4 ***p*<0.01, WT mice vs VGLUT3^T8I/T8I^ mice). **h,** Non-reinforced active nose-pokes during the 2 h last extinction session in 10 minutes blocks for WT and VGLUT^T8I/T8I^ mice, no cue-light or cocaine infusion was administered. **i**, Non-reinforced active nose-pokes during the 2 h cue-induced reinstatement session showing the number of responses in 10 minutes blocks for WT and VGLUT^T8I/T8I^ mice. No cocaine infusion was administered contingent on the response and the cue light (genotype x time, F11,253=2.6284, *p*<0.01, two-way ANOVA repeated measures, and LSD post hoc test 70 minutes ****p*<0.001, 80 minutes ****p*<0.001, 90 minutes ****p*<0.05 and 100 minutes ***p*<0.05, WT mice vs VGLUT3^T8I/T8I^ mice). Source data are provided as a Source Data file.

**
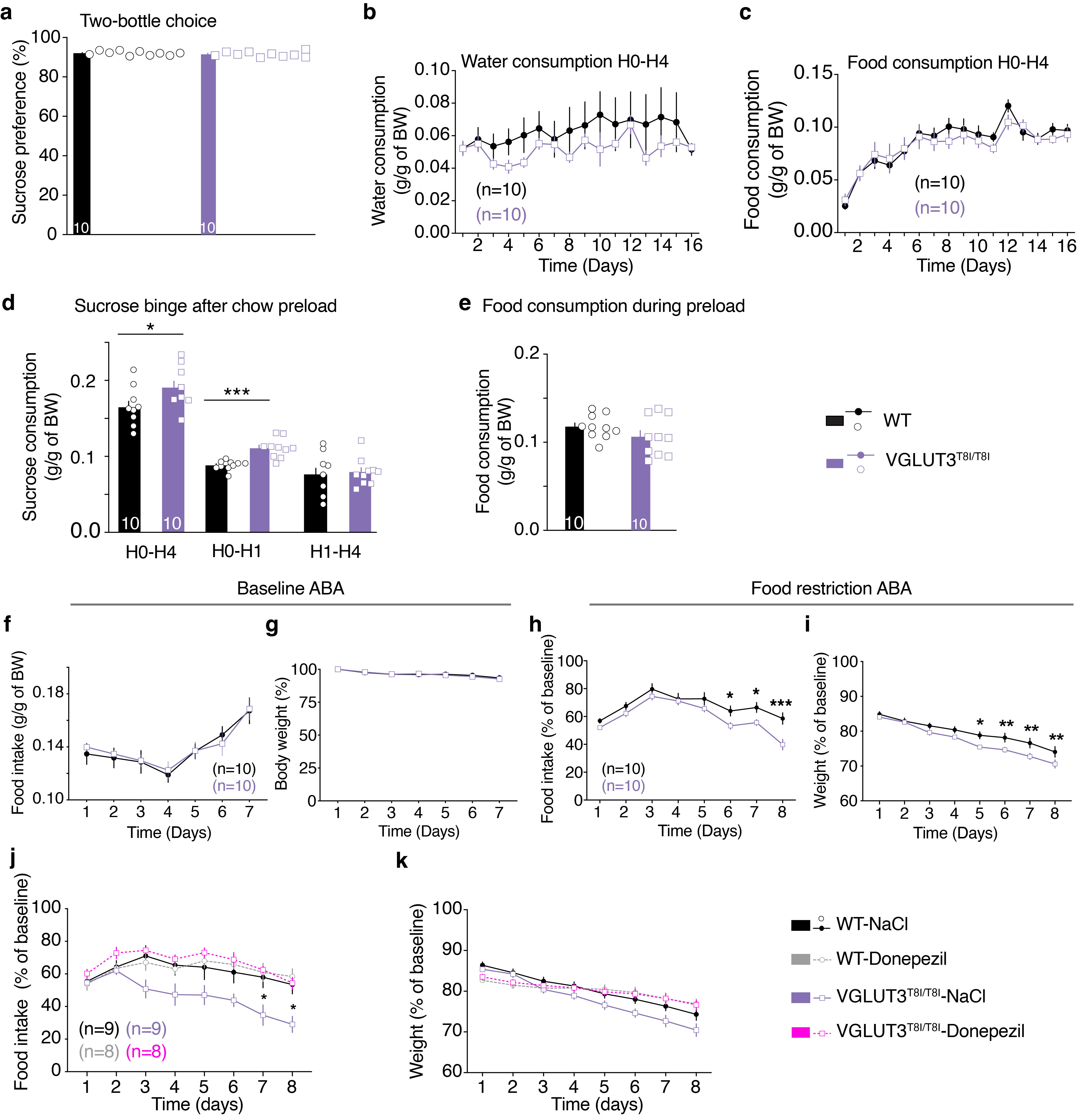
**

**Supplementary Fig. 4 Binge-like sucrose overconsumption and activity-based anorexia models. a**, Sucrose preference measured by the two-bottle choice test. **b**-**d**, Sucrose binge-like overconsumption test. **b**, Daily water consumption during the 4 hours of sucrose access (H0-H4). **c**, Daily food consumption during the 4 hours of sucrose access (H0-H4). **d**, Sucrose consumption after 1-hour ad libitum food exposure during H0-H4, H0-H1 and H1-H4. **e**, Food consumption during the 1-hour ad libitum food exposure. **f**-**k**, Activity-based anorexia (ABA) model. **f**, Food intake (**f**) and body weight (**g**) during the baseline of ABA. Food intake (**h**) and body weight (**i**) during the food-restriction period of ABA. **j**,**k** Effect of chronic donepezil (intraperitoneal IP, 0.3 mg.kg^-1^) treatment on food intake (**j**) and on body weight (**k**) of WT mice or VGLUT3^T8I/T8I^ mice. Statistical analysis was performed with two-tailed unpaired t-test (**a**,**d**,**e)**, two-way repeated-measures ANOVA (**b**,**c**,**f**-**k**). Source data are provided as a Source Data file.

**2) Within-cases analyses**

cocaine - alcohol - opioid - benzodiazepine - cannabis dependence, total score on the SAPS-CIP and for each of four subscales (hallucinations, delusions, stereotypies, agitation)

**2a) Rare missense variants:** VGLUT3 gene p.T8I *vs.* VGLUT1&2 variants *vs.* none

***All ancestries. P.T8I carriers***

*CUD cases =8*

*Controls (gnomAD) =1,674*

**Initial sample**

*581 participants genotyped on two waves, merged on 566,392 markers*

**Quality control, *PLINK2***

- Relatedness (Identity-by-Descent): 34 excluded
- Hardy-Weinberg equilibrium at *p* <1e-6: 100% OK
- Missing genotypes >2%: 2 excluded
- Sex: 100% OK
- 6 principal components of ancestry computed

*N=524 ; 525,681 markers*

**Candidate genes: *SLC17A6, SLC1717, SLC17A8***

34 variants on the DNA array

**1) Case-control analysis**

**2b) Frequent variants**

**Supplementary Fig. 5 SUDs sample flowchart.**


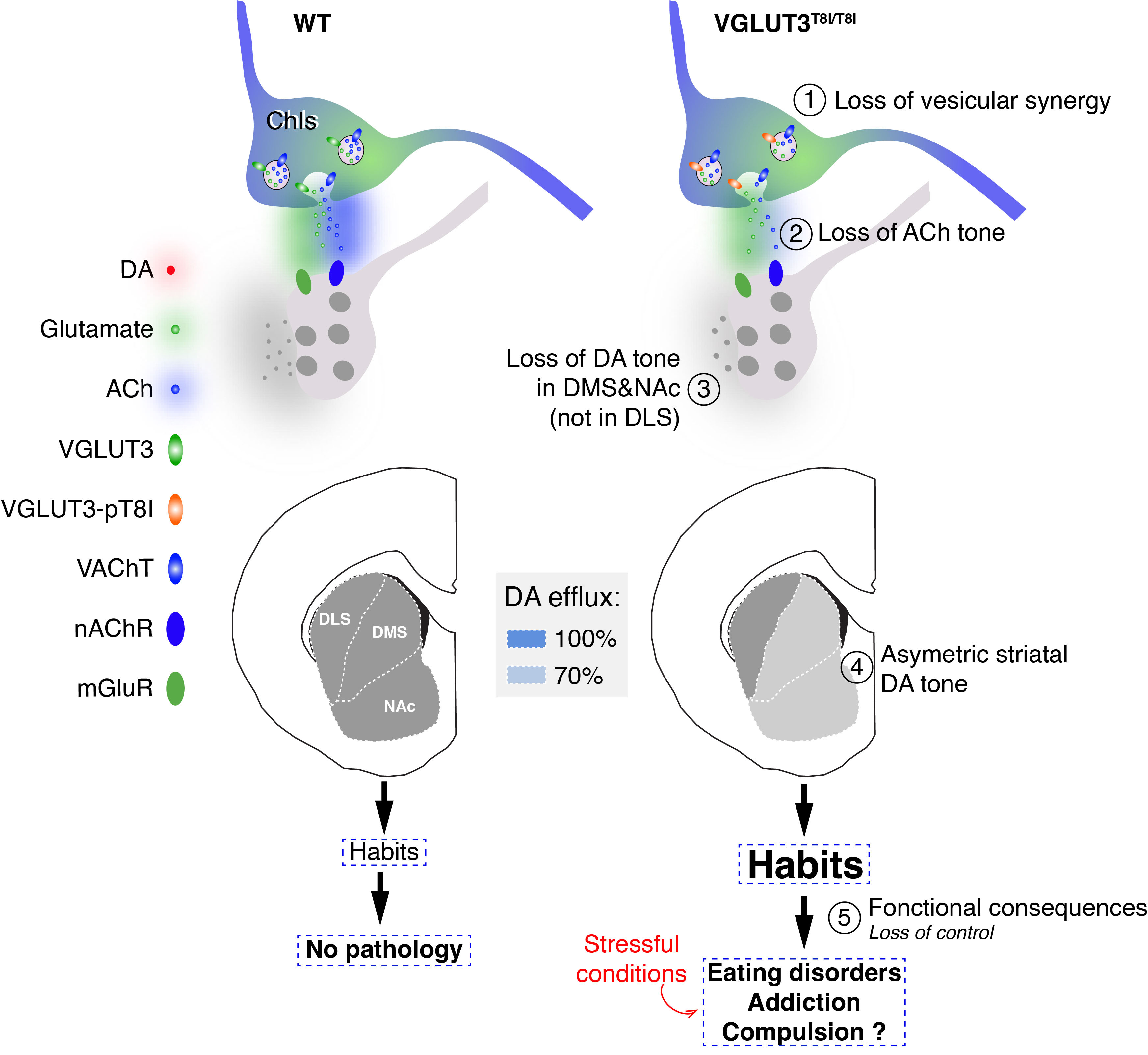


**Supplementary Fig. 6 Hypothetical model.** The presence of VGLUT3 provides ChIs with the ability to signal with glutamate and increase vesicular ACh accumulation. The VGLUT3-pT8I mutation blunts vesicular synergy (1) without disrupting glutamate transport. Consequently, ACh release is reduced (2) in the striatum of VGLUT3^T8I/T8I^ mice. Striatal ACh stimulates DA release in the nucleus accumbens (NAc) and the DMS (caudate in humans) but not in the DLS (putamen in humans) of rodents^9, 10, 11^. In VGLUT3^T8i/T8i^ mice, imbalanced DA levels in the DMS relative to the DLS (3 and 4) lead to rapid transitions from goal-directed behaviors to habits. Under control conditions, VGLUT3^T8i/T8i^ mice feed normally, but stressful conditions (e.g., restricted access to food) precipitate maladaptive eating behaviors in mutant mice (5). Based on these findings, it can be hypothesized that imbalanced striatal DA transmission could lead not only to EDs but also to SUDs and other compulsive disorders.

**Table S10: Statistics for manuscript page 7 and for Supplementary Fig. S1**

| *Distribution and frequency comparison of cocaine routes of administration (injection and smoking* vs. *snorting) as a function of the presence of p.T8I. Total n=351* | |
| --- | --- |
| Cocaine injection and smoking :  T8I, n=1 (1%); noT8I, n =124 (99%)  Cocaine snorting:  T8I, n=2 (1%); noT8I, n =224 (99%) | Fisher's Exact Test p-value=1 |
|  | |
| *Bivariate associations between VGLUT3 gene variants and phenotypic variables in patients with addictive disorders. N =338 - SAPS scores not imputed.* (Supplementary Fig. 1a) | |
| Kruskal-Wallis test of SAPS total score between patients with p.T8I variant (n=2), other VGLUT3 variants (n=4) and no VGLUT3 variant (n=332) | |
| p.T8I vs. other SLC17A8 vs. no SLC17A8 mutation | |
| SAPS scores [median (IQR)]:  p.T8I, 11 (11-11)  Other SLC17A8 variants, 3 (2.3-3.5)  No SLC17A8 variants, 6 (3-9) | Kruskal-Wallis chi-squared= 6.8838, df=2,  p-value=0.032  Effect size=0.0136 (small) |

**Table S11: Statistics for Supplementary Fig. 2**

| ACh fiber photometry - Median (Supplementary Fig. S2a) | |
| --- | --- |
| Unpaired t-test - WT mice *vs* VGLUT3^T8i/T8i^ mice | |
| n | p-value |
| 7-7 | 0.868 |
| Wilcoxon rank sum test (non-paired) | |
| 7-7 | 0.535  (W=19) |

| ACh fiber photometry - Treshold (Supplementary Fig. S2c) | |
| --- | --- |
| Unpaired t-test - WT mice *vs* VGLUT3^T8i/T8i^ mice | |
| n | p-value |
| 7-7 | 0.584 |
| Wilcoxon rank sum test (non-paired) | |
| 7-7 | 0.535  (W=19) |

**Table S12: Statistics for Supplementary Fig. 3**

| Anxiety (O-Maze) - number of entries (Supplementary Fig. S3a) | |
| --- | --- |
| Unpaired t-test - WT mice *vs* VGLUT3^T8i/T8i^ mice (n=12-12) | |
|  | p-value |
| Closed arms | 0.523 |
| Open arms | 0.536 |

| Anxiety (O-Maze) - time in open arms (%) (Supplementary Fig. S3b) | |
| --- | --- |
| Unpaired t-test - WT mice *vs* VGLUT3^T8i/T8i^ mice | |
| n | p-value |
| 12-12 | 0.977 |

| Anxiety (light/dark box) - Latency to enter the light compartment - (Supplementary Fig. S3c) | |
| --- | --- |
| Unpaired t-test - WT mice *vs* VGLUT3^T8i/T8i^ mice | |
| n | p-value |
| 26-17 | 0.257 |

| Anxiety (light/dark box) – Number of transition - (Supplementary Fig. S3d) | |
| --- | --- |
| Unpaired t-test - WT mice *vs* VGLUT3^T8i/T8i^ mice | |
| n | p-value |
| 26-17 | 0.265 |

| Anxiety (light/dark box) – Time in light-zone- (Supplementary Fig. S3e) | |
| --- | --- |
| Unpaired t-test - WT mice *vs* VGLUT3^T8i/T8i^ mice | |
| n | p-value |
| 26-17 | 0.426 |

| Cocaine self-administration - inactive nose-poke (Supplementary Fig. S3f) | | |
| --- | --- | --- |
|  | WT mice *vs* VGLUT3^T8I/T8I^ mice (n=14-11) | |
|  | Two-way ANOVA repeated measures | |
|  | F-value | p-value |
| Genotype | F_1,23_=0.1544 | 0.6980 |
| Time | F_9,207_=0.564 | <0.01 |
| Genotype x Time | F_9,207_=0.2701 | 0.9820 |

| Cocaine self-administration - infusions (Supplementary Fig. S3g) | | |
| --- | --- | --- |
|  | WT mice *vs* VGLUT3^T8I/T8I^ mice (n=14-11) | |
|  | Two-way ANOVA repeated measures | |
|  | F-value | p-value |
| Genotype | F_1,23_=2.4224 | 0.1333 |
| Time | F_9,207_=2.4963 | <0.01 |
| Genotype x Time | F_9,207_=2.0852 | <0.05 |
| LSD posthoc | WT vs VGLUT3^T8I/T8I^ mice | <0.05 (day3) |
| LSD posthoc | WT vs VGLUT3^T8I/T8I^ mice | <0.01 (day 4) |

| Cocaine self-administration - inactive nose-poke (Supplementary Fig. S3h) | | |
| --- | --- | --- |
|  | WT mice *vs* VGLUT3^T8I/T8I^ mice (n=14-11) | |
|  | Two-way ANOVA repeated measures | |
|  | F-value | p-value |
| Genotype | F_1,23_=0.1544 | 0.6980 |
| Time | F_9,207_=0.564 | <0.01 |
| Genotype x Time | F_9,207_=0.2701 | 0.9820 |

| Cocaine self-administration – Last extinction session in 10 minutes blocks (Supplementary Fig. S3h) | | |
| --- | --- | --- |
|  | WT mice *vs* VGLUT3^T8I/T8I^ mice (n=14-11) | |
|  | Two-way ANOVA repeated measures | |
|  | F-value | p-value |
| Genotype | F_1,23_=2.2075 | 0.1509 |
| Time | F_11,253_=0.9568 | 0.4865 |
| Genotype x Time | F_11,253_=0.9851 | 0.4603 |

| Cocaine self-administration - infusions (Supplementary Fig. S3i) | | |
| --- | --- | --- |
|  | WT mice *vs* VGLUT3^T8I/T8I^ mice (n=14-11) | |
|  | Two-way ANOVA repeated measures | |
|  | F-value | p-value |
| Genotype | F_1,23_=2.4224 | 0.1333 |
| Time | F_9,207_=2.4963 | <0.01 |
| Genotype x Time | F_9,207_=2.0852 | <0.05 |
| LSD posthoc | WT vs VGLUT3^T8I/T8I^ mice | 0.9818 (day 1) |
| LSD posthoc | WT vs VGLUT3^T8I/T8I^ mice | 0.2534 (day 2) |
| LSD posthoc | WT vs VGLUT3^T8I/T8I^ mice | <0.05 (day 3) |
| LSD posthoc | WT vs VGLUT3^T8I/T8I^ mice | <0.01 (day 4) |
| LSD posthoc | WT vs VGLUT3^T8I/T8I^ mice | 0.0843 (day 5) |
| LSD posthoc | WT vs VGLUT3^T8I/T8I^ mice | 0.1294 (day 6) |
| LSD posthoc | WT vs VGLUT3^T8I/T8I^ mice | 0.3839 (day 7) |
| LSD posthoc | WT vs VGLUT3^T8I/T8I^ mice | 0.8477 (day 8) |
| LSD posthoc | WT vs VGLUT3^T8I/T8I^ mice | 0.7010 (day 9) |
| LSD posthoc | WT vs VGLUT3^T8I/T8I^ mice | 0.6021 (day 10) |

| Cocaine self-administration – Last extinction session in 10 minutes blocks (Supplementary Fig. S3h) | | |
| --- | --- | --- |
|  | WT mice *vs* VGLUT3^T8I/T8I^ mice (n=14-11) | |
|  | Two-way ANOVA repeated measures | |
|  | F-value | p-value |
| Genotype | F_1,23_=2.2075 | 0.1509 |
| Time | F_11,253_=0.9568 | 0.4865 |
| Genotype x Time | F_11,253_=0.9851 | 0.4603 |

**Table S13: Statistics for Supplementary Fig. 4**

| Sucrose preference - Two-bottle choice test (Supplementary Fig. S4a) | |
| --- | --- |
| Unpaired t-test - WT mice *vs* VGLUT3^T8i/T8i^ mice | |
| n | p-value |
| 10-10 | 0.341 |

| Sucrose binge-like overconsumption model - Water intake H0-H4 (Supplementary Fig. S4b) | | |
| --- | --- | --- |
|  | WT mice *vs* VGLUT3^T8i/T8i^ mice (n=10-10) | |
|  | Two-way ANOVA repeated measures | |
|  | F-value | p-value |
| Genotype | F_1,18_=0.828 | 0.378 |
| Time | F_15,270_=2.198 | 0.007 |
| Genotype x Time | F_15,270_=0.903 | 0.561 |

| Sucrose binge-like overconsumption model - Food intake H0-H4 (Supplementary Fig. S4c) | | |
| --- | --- | --- |
|  | WT mice *vs* VGLUT3^T8i/T8i^ mice (n=10-10) | |
|  | Two-way ANOVA repeated measures | |
|  | F-value | p-value |
| Genotype | F_1,18_=0.128 | 0.725 |
| Time | F_15,270_=30.43 | <0.001 |
| Genotype x Time | F_15,270_=0.979 | 0.477 |

| Sucrose binge-like overconsumption model - Sucrose intake after chow preload (Supplementary Fig. S4d) | | |
| --- | --- | --- |
| Unpaired t-test - WT mice *vs* VGLUT3^T8i/T8i^ mice | | |
|  | n | p-value |
| H0-H4 | 10-10 | 0.037 |
| H0-H1 | 10-10 | <0.001 |
| H1-H4 | 10-10 | 0.752 |

| Sucrose binge-like overconsumption model - Food intake during chow preload  (Supplementary Fig. S4e) | |
| --- | --- |
| Unpaired t-test - WT mice *vs* VGLUT3^T8i/T8i^ mice | |
| n | p-value |
| 10-10 | 0.179 |

| Activity based anorexia model - Food intake during baseline (Supplementary Fig. S4f) | | |
| --- | --- | --- |
|  | WT mice *vs* VGLUT3^T8i/T8i^ mice (n=10-10) | |
|  | Two-way ANOVA repeated measures | |
|  | F-value | p-value |
| Genotype | F_1,18_=0.02 | 0.887 |
| Time | F_2.087,37.56_=20.87 | <0.001 |
| Genotype x Time | F_6,108_=0.336 | 0.917 |

| Activity based anorexia model – Body weight during baseline (Supplementary Fig. S4g) | | |
| --- | --- | --- |
|  | WT mice *vs* VGLUT3^T8i/T8i^ mice (n=10-10) | |
|  | Two-way ANOVA repeated measures | |
|  | F-value | p-value |
| Genotype | F_1,18_=0.041 | 0.841 |
| Time | F_2.165,38.96_=39.15 | <0.001 |
| Genotype x Time | F_6,108_=0.726 | 0.63 |

| Activity based anorexia model - Food intake during the food-restriction period  (Supplementary Fig. S4h) | | |
| --- | --- | --- |
|  | WT mice *vs* VGLUT3^T8i/T8i^ mice (n=10-10) | |
|  | Two-way ANOVA repeated measures | |
|  | F-value | p-value |
| Genotype | F_1,18_=5.437 | 0.032 |
| Time | F_7,126_=42.55 | <0.001 |
| Genotype x Time | F_7,126_=3.526 | 0.002 |

| Activity based anorexia model – Body weight during the food-restriction period  (Supplementary Fig. S4i) | | |
| --- | --- | --- |
|  | WT mice *vs* VGLUT3^T8i/T8i^ mice (n=10-10) | |
|  | Two-way ANOVA repeated measures | |
|  | F-value | p-value |
| Genotype | F_1,18_=7.364 | 0.014 |
| Time | F_7,126_=63.12 | <0.001 |
| Genotype x Time | F_7,126_=1.716 | 0.111 |

| Activity based anorexia model – Food intake during the food-restriction period  - Treatment with Donepezil (Supplementary Fig. S4j) | | |
| --- | --- | --- |
|  | WT mice-NaCl *vs* WT mice-Donepezil *vs* VGLUT3^T8i/T8i^ mice-NaCl *vs* VGLUT3^T8i/T8i^ mice-Donepezil (n=9-8-9-8) | |
|  | Two-way ANOVA repeated measures | |
|  | F-value | p-value |
| Group | F_3,30_=5.484 | 0.004 |
| Time | F_4.146,124.4_=18.03 | <0.001 |
| Group x Time | F_21,210_=3.281 | <0.001 |

| Activity based anorexia model - Body weight during the food-restriction period  - Treatment with Donepezil (Supplementary Fig. S4k) | | |
| --- | --- | --- |
|  | WT mice-NaCl *vs* WT mice-Donepezil *vs* VGLUT3^T8i/T8i^ mice-NaCl *vs* VGLUT3^T8i/T8i^ mice-Donepezil (n=9-8-9-8) | |
|  | Two-way ANOVA repeated measures | |
|  | F-value | p-value |
| Group | F_3,30_=1.956 | 0.142 |
| Time | F_7,210_=93.24 | <0.001 |
| Group x Time | F_21,210_=5.985 | <0.001 |

**Supplementary references**

1. Fairburn C, Cooper P. The Eating Disorders Examination. In: *Binge Eating: Nature, Assessment and Treatment.* (ed C. Fairburn GW). The Guilford Press (1993).

2. Mouly S*, et al.* Methadone dose in heroin-dependent patients: role of clinical factors, comedications, genetic polymorphisms and enzyme activity. *Br J Clin Pharmacol* **79**, 967-977 (2015).

3. Vorspan F*, et al.* Self-reported cue-induced physical symptoms of craving as an indicator of cocaine dependence. *Am J Addict* **24**, 740-743 (2015).

4. Association AP. *Diagnostic and Statistical Manual of Mental Disorders, 4th Edition, Text Revision (DSM-IV-TR)*. Amer Psychiatric Pub Inc, 1987 (2013).

5. Marees AT*, et al.* A tutorial on conducting genome-wide association studies: Quality control and statistical analysis. *Int J Methods Psychiatr Res* **27**, e1608 (2018).

6. McLellan AT*, et al.* The Fifth Edition of the Addiction Severity Index. *J Subst Abuse Treat* **9**, 199-213 (1992).

7. Gutierrez-Cuesta J, Burokas A, Mancino S, Kummer S, Martin-Garcia E, Maldonado R. Effects of genetic deletion of endogenous opioid system components on the reinstatement of cocaine-seeking behavior in mice. *Neuropsychopharmacology* **39**, 2974-2988 (2014).

8. Martin-Garcia E*, et al.* New operant model of reinstatement of food-seeking behavior in mice. *Psychopharmacology (Berl)* **215**, 49-70 (2011).

9. Brimblecombe KR, Threlfell S, Dautan D, Kosillo P, Mena-Segovia J, Cragg SJ. Targeted Activation of Cholinergic Interneurons Accounts for the Modulation of Dopamine by Striatal Nicotinic Receptors. *eNeuro* **5**, (2018).

10. Favier M*, et al.* Cholinergic dysfunction in the dorsal striatum promotes habit formation and maladaptive eating. *J Clin Invest* **130**, 6616-6630 (2020).

11. Sakae DY*, et al.* The absence of VGLUT3 predisposes to cocaine abuse by increasing dopamine and glutamate signaling in the nucleus accumbens. *Mol Psychiatry* **20**, 1448-1459 (2015).
