# Supplementary material for: The human VGLUT3-pT8I mutation elicits uneven striatal DA signaling, food or drug maladaptive consumption in male mice
Source: Nat Commun. 2024 Jul 7;15:5691. doi: 10.1038/s41467-024-49371-1 (PMC11227582; doi:10.1038/s41467-024-49371-1)
Supplement: Supplementary file 3 — Description of Additional Supplementary Information [file 41467_2024_49371_MOESM3_ESM.pdf]

Description of Additional Supplementary Files

Supplementary Dataset 1: description of all frequent single nucleotide polymorphisms from the *SLC17A8* gene analyzed in the study after imputation. RsID, variant name according to dbSNP version 150; POS, position on chromosome 12 (base pairs); ref, reference allele; alt, risk allele; minor allele frequencies in the 1000 genomes population =Africans (AFR), Mixed Americans (AMR), South-East Asians (EAS), Europeans (EUR), South Asians (SAS); consequence according to ENSEMBL v.102 annotation; SIFT, Sorting Intolerant from Tolerant ; PolyPhen, Polymorphism Phenotyping ; CADD, Combined Annotation Dependent Depletion
