# Supplementary material for: The human VGLUT3-pT8I mutation elicits uneven striatal DA signaling, food or drug maladaptive consumption in male mice
Source: Nat Commun. 2024 Jul 7;15:5691. doi: 10.1038/s41467-024-49371-1 (PMC11227582; doi:10.1038/s41467-024-49371-1)
Supplement: Supplementary file 5 — Reporting Summary [file 41467_2024_49371_MOESM5_ESM.pdf]

Corresponding author(s): Salah El Mestikawy & Mathieu Favier

Last updated by author(s): 2024/04/18

Reporting Summary

Nature Portfolio wishes to improve the reproducibility of the work that we publish. This form provides structure for consistency and transparency in reporting. For further information on Nature Portfolio policies, see our Editorial Policies and the Editorial Policy Checklist.

Statistics

For all statistical analyses, confirm that the following items are present in the figure legend, table legend, main text, or Methods section.

n/a	Confirmed	
☐	☒	The exact sample size (*n*) for each experimental group/condition, given as a discrete number and unit of measurement
☐	☒	A statement on whether measurements were taken from distinct samples or whether the same sample was measured repeatedly
☐	☒	The statistical test(s) used AND whether they are one- or two-sided<br>*Only common tests should be described solely by name; describe more complex techniques in the Methods section.*
☒	☐	A description of all covariates tested
☐	☒	A description of any assumptions or corrections, such as tests of normality and adjustment for multiple comparisons
☐	☒	A full description of the statistical parameters including central tendency (e.g. means) or other basic estimates (e.g. regression coefficient) AND variation (e.g. standard deviation) or associated estimates of uncertainty (e.g. confidence intervals)
☐	☒	For null hypothesis testing, the test statistic (e.g. *F*, *t*, *r*) with confidence intervals, effect sizes, degrees of freedom and *P* value noted<br>*Give P values as exact values whenever suitable.*
☐	☒	For Bayesian analysis, information on the choice of priors and Markov chain Monte Carlo settings
☒	☐	For hierarchical and complex designs, identification of the appropriate level for tests and full reporting of outcomes
☐	☒	Estimates of effect sizes (e.g. Cohen's *d*, Pearson's *r*), indicating how they were calculated

Our web collection on statistics for biologists contains articles on many of the points above.

Software and code

Policy information about availability of computer code

Data collection	For both animal and human studies data were collected manually or automatically by commercially available sources associated with respective equipments as indicated in Material and Methods. No custom codes were used for data collection.
Data analysis	Human data were analyzed using R and R studio. Code is available as supplementary file.<br><br>3D modelization of VGLUT3-p.T8I was performed with the package from Biovia Discovery studio (Dassault Systemes), equilibrated using a short Nanoscale Molecular Dynamic software program (NAMD) of 1 ns.<br><br>Immunoautoradiographic data were analyzed with MCID analysis software (InterFocus, Ltd).<br><br>Autapses electrophysiological recording Current traces were analyzed using Axograph X, Excel (Microsoft), and Prism (GraphPad Software). The mEPSCs were detected with a template function (Axograph; template: rise, 0.5 ms; decay, 3 ms; criteria range: rise, 0.15–1.5 m; decay, 0.5–5 ms).<br><br>In vivo fiber photometry of ACh data were analyzed on R software. Custom code used to analyze fiber photometry are available upon request to Alexandre Mourot (alexandre.mourot@espci.psl.eu).<br><br>In vivo voltammetry of DA release data were collected with FAST (Quanteon System 3, Quanteon, Lexington, Kentucky, USA). Data were then converted to Microsoft Excel (16.83) and analyzed with GraphPad Prism (10.2.2).

Behavioral experiments data were converted to Microsoft Excel (16.83) and analyzed with GraphPad Prism (10.2.2).

For manuscripts utilizing custom algorithms or software that are central to the research but not yet described in published literature, software must be made available to editors and reviewers. We strongly encourage code deposition in a community repository (e.g. GitHub). See the Nature Portfolio guidelines for submitting code & software for further information.

Data

Policy information about availability of data

All manuscripts must include a data availability statement. This statement should provide the following information, where applicable:

- Accession codes, unique identifiers, or web links for publicly available datasets
- A description of any restrictions on data availability
- For clinical datasets or third party data, please ensure that the statement adheres to our policy

Data availability: For clinical data and human genetic individual-level genotypes cannot be made available for ethical reasons. Summary statistics and data underlying the figures are available upon request to Romain Icick (romain.icick@aphp.fr). Raw data from all molecular, cellular or mouse investigations are available without restrictions upon request to Salah El Mestikawy (salah.elmestikawy@mcgill.ca). All unique biological materials (knock-out mice or antiserums) used are readily available upon request to Salah El Mestikawy. Matlab custom code for acetylcholine fiber photometry are available upon request to Alexandre Mourot (alexandre.mourot@espci.psl.eu). Source data are provided with this paper.

Research involving human participants, their data, or biological material

Policy information about studies with human participants or human data. See also policy information about sex, gender (identity/presentation), and sexual orientation and race, ethnicity and racism.

Reporting on sex and gender	For human studies, consent was obtained from all participants. Sex (biological attribute) of human participants is indicated in the manuscript and was determined on a self-reported declarative basis as well as on a genetically basis. Sex was considered in the study design.
Reporting on race, ethnicity, or other socially relevant groupings	Sociodemographic data including race and income were part of the initial characterization of the population. However, they were not used as confounders since the subgroups were very small due to the characteristics of the variants studied. Regarding race and ethnicity, we focused on genetically-informed ancestry instead.
Population characteristics	We investigated the phenotypic correlates of SLC17A8 variants in two independent samples of genotyped patients and healthy controls who each underwent extensive characterization for addictive and mental disorders. These samples were chosen since their participants were recruited for disorders showing a strong compulsive component, which likely represents a transdiagnostic endophenotype underlying the vulnerability to several mental disorders.<br>- Sample #1 eating disorders (EDs sample), recruited by the Douglas hospital eating disorders program, including healthy controls women (n=71) and 270 women out- and inpatients with diagnosis for anorexia nervosa (n=74) bulimia nervosa (n=116), eating disorders not otherwise specified (EDNOS, n=80), mean age=25±0.4 year-old.<br>- - Sample #2 substance used disorders (SUDs sample), recruited in specialized SUDs outpatient clinics 524 outpatients (77% men) seeking treatment for SUDs, mean age=43±9-year-old, genotyped by DNA array f<br>Sample #2 substance used disorders (SUDs sample), recruited in specialized SUDs outpatient clinics 524 outpatients (77% men) seeking treatment for SUDs, mean age=43±9-year-old, genotyped by DNA array for 550,000+ rare and frequent genetic variants.
Recruitment	- Sample#1 eating disorders (ED sample) recruited by the Douglas eating disorders program between 2000 and 2009. Information are available upon request to Howard Steiger (howard.steiger.COMTL@ssss.gouv.qc.ca).<br>- Sample#2 substance use disorders (SUD sample) recruited in specialized SUDs outpatient clinics (N=524 genotyped) between 2008 and 2014. All patients, seeking treatment for problematic cocaine use were asked to participate if they did not show exclusion criteria. Note: the addiction setting sees about 150 new patients with problematic cocaine use per year. Therefore, about 50% of all patients were included.
Ethics oversight	- ED sample: all participants provided written informed consent and all procedures contributing to this work complied with the ethical standards of the relevant national and institutional committees (Douglas Institute, McGill University) on human experimentation.<br><br>- SUD sample: the study protocols were approved by the local ethics committees (CPP Ile-de-France VI for study one and CPP Ile de France IV for study two) and preregistered (clinicaltrials.gov NCT00894452 and NCT01569347, respectively), and by the relevant Institutional Review Board for further analyses of the combined sample [CEEI from the Institut de la Santé et de la Recherche Médicale (INSERM), IRB00003888 in July 2015]. All participants provided written informed consent, and study records were continuously monitored by the local research administration (Unité de Recherche Clinique) to ensure their conformity to the original protocols.<br><br>- Authors assert that all procedures contributing to this work comply with the ethical standards of the relevant national and institutional committees on human experimentation and with the Helsinki Declaration of 1975, as revised in 2008.

Note that full information on the approval of the study protocol must also be provided in the manuscript.

Field-specific reporting

Please select the one below that is the best fit for your research. If you are not sure, read the appropriate sections before making your selection.

☐ Life sciences ☒ Behavioural & social sciences ☐ Ecological, evolutionary & environmental sciences

For a reference copy of the document with all sections, see nature.com/documents/nr-reporting-summary-flat.pdf

Behavioural & social sciences study design

All studies must disclose on these points even when the disclosure is negative.

Study description	In this study we used human genetic and clinical data that were quantitative.<br>We also used primary neuronal culture and autapses to conduct anatomical and electrophysiological characterizations. A mutant mouse line was used to conduct molecular, biochemical (in vivo voltammetry and in vivo fiber photometry) and behavioral studies.<br>All animal data within this manuscript are quantitative experimental.
Research sample	We investigated the phenotypic correlates of SLC17A8 variants in two independent samples of genotyped patients and healthy controls who each underwent extensive characterization for addictive and mental disorders. These samples were chosen since their participants were recruited for disorders showing a strong compulsive component, which likely represents a transdiagnostic endophenotype underlying the vulnerability to several mental disorders.<br>A total of 235 male mice (2-8 months-old, 121 WT mice and 114 VGLUT3T8I/T8I mice) were used. The sample size is representative and was chosen based on our previous (see Favier et al., 2020 Journal of Clinical Investigations).
Sampling strategy	All sample sizes were chosen according to comparable type of experiments in the literature and based on our previous publication (see Favier et al., 2020 Journal of Clinical Investigations).<br>For mice, our studies were exploratory, and the size effect was not known “à priori”. Groups of 10-15 animals were used.<br>For the human study, there was no particular sampling strategy, since samples were already fully biased toward severe mental disorders. In neither the clinical settings was any further sampling applied, so that recruitment was consecutive during the studies periods.
Data collection	All data were collected using computers. Whenever possible, the investigator was blinded during experimental procedures (this was the case for all anatomical, measurements and voltammetry or fiber photometry experiments). When behavioral experiments imposed the investigator not to be blinded, to avoid biases mice were allocated randomly to experimental groups and experimental conditions were counterbalanced across groups.<br>Researchers were blind to the group of patients (eating disorder and substance use disorder patients/controls).
Timing	Data were collected between 2016/09 and 2022/09.
Data exclusions	No data were excluded, except for:<br><br>- [3H]Acetylcholine vesicular uptake : one Glu+ experiment was discarded for technical reason (clogged filters).<br>- Cocaine self-administration where 2 WT mice and 2 VGLUT3T8I/T8I mice were excluded: animals which did not perform cocaine self-administration were excluded (see figure 4G acquisition %).<br>- Devaluation tests where 1 WT mouse and 3 VGLUT3T8I/T8I mice were excluded: mice that did not consume a minimum of 0.4g of food were not included in the analysis as classically done for this type of experiments (see Rossi et al., 2012).
Non-participation	Pr Howard Steiger (Eating disorders clinic, Montreal QC, Canada) dropped out and declined participation.
Randomization	Allocation into experimental groups was randomized.

Reporting for specific materials, systems and methods

We require information from authors about some types of materials, experimental systems and methods used in many studies. Here, indicate whether each material, system or method listed is relevant to your study. If you are not sure if a list item applies to your research, read the appropriate section before selecting a response.

Materials & experimental systems

n/a	Involved in the study	
☐	☒	Antibodies
☒	☐	Eukaryotic cell lines
☒	☐	Palaeontology and archaeology
☐	☒	Animals and other organisms
☐	☒	Clinical data
☒	☐	Dual use research of concern
☒	☐	Plants

Methods

n/a	Involved in the study	
☒	☐	ChIP-seq
☒	☐	Flow cytometry
☒	☐	MRI-based neuroimaging

Antibodies

Antibodies used

Antiserums used in this study are either from commercial source or "home-made" (see references) and available upon request to Salah El Mestikawy.

- Anti-human VGLUT3 rabbit polyclonal antiserum (home-made, dilution1:1000). Reference : Vigneault E, et al. Distribution of vesicular glutamate transporters in the human brain. Front Neuroanat 9, 23 (2015).
- Anti-rodent VGLUT1 rabbit polyclonal antiserum (home-made, dilution 1:2000). Reference : Herzog E, et al. The existence of a second vesicular glutamate transporter specifies subpopulations of glutamatergic neurons. J Neurosci 21, RC181 (2001).
- Anti-VAChT Guinea pig polyclonal (home-made, dilution1:5000). Reference: Gras C, et al. The vesicular glutamate transporter VGLUT3 synergizes striatal acetylcholine tone. Nat Neurosci 11, 292-300 (2008).
- Anti-rodent VGLUT3 Rabbit polyclonal (Synaptic System, Goettingen, Germany, ref 135–203/26. Dilution 1:20,000).
- Anti-rodent VGLUT3 rabbit polyclonal antiserum (Synaptic System, Goettingen, Germany, ref 135–203/26. Dilution 1:2000).
- Anti-VAChT Rabbit polyclonal (Synaptic System, Goettingen, Germany, ref 139-103/34. Dilution 1:5000)
- Anti-rodent MAP2 mouse monoclonal antiserum (Sigma-Aldrich ref M9942 clone HM-2, purified from hybridoma cell culture. Dilution 1:1000).
- Anti-Bassoon/BSN antibody mouse monoclonal (Abcam [SAP7F407] (ab82958). Dilution 1:2000).
- Anti-PSD95 antibody mouse monoclonal [K28/43] - (Abcam Synaptic Marker (ab192757) Mouse monoclonal [K28/43]. Dilution 1:2000).
- Alexa Fluor 488 Donkey anti-rabbit IgG (purified) secondary antibody (ThermoFisher scientific, Cat # A-21206)
- Alexa Fluor 555 Donkey anti-rabbit IgG (purified) secondary antibody (ThermoFisher scientific, Cat # A-31572

Validation

Validation of home-made antiserums:
Home-made anti-rodent and anti-human VGLUT3 rabbit polyclonal antiserum, anti-rodent and anti-VAChT Guinea pig polyclonal antiserum were validated against corresponding knockout mouse line. Anti-VGLUT1 rabbit polyclonal antiserum was validated by western blot, immunoautoradiography and immunofluorescence after blocking specific binding sites with corresponding antigenic peptide (see Herzog et al., 2001, PMID 11698619).
These antiserums are all suitable for western blot, immunoautoradiography, immunohistochemistry, and immunofluorescence.
Validation of commercial antiserums by provider:
- Anti-rodent VGLUT3 Rabbit polyclonal (Synaptic System, Goettingen, Germany). Affinity purified and validated with VGLUT3-KO mice. Suitable applications: western blot, immunochemistry, immunofluorescence.
- Anti-VAChT Rabbit polyclonal (Synaptic System, Goettingen, Germany). Affinity purified and validated with VAChT-KO mice. Suitable applications: western blot, immunochemistry, immunofluorescence, immunoprecipitation.
- Anti-rodent MAP2 mouse monoclonal antiserum (Sigma-Aldrich). Validated by immunoblotting, immunofluorescence.
- Anti-Bassoon/BSN antibody mouse monoclonal (Abcam [SAP7F407] (ab82958). Dilution 1:2000). Validated by immunoblotting, immunofluorescence, immunohistochemistry.
- Anti-PSD95 antibody mouse monoclonal [K28/43] - (Abcam Synaptic Marker (ab192757) Mouse monoclonal [K28/43]. Dilution 1:2000). Validated by western blotting, immunofluorescence, immunohistochemistry.

Animals and other research organisms

Policy information about studies involving animals; ARRIVE guidelines recommended for reporting animal research, and Sex and Gender in Research

Laboratory animals

A mouse line expressing the p.T8I point mutation in the Slc17a8 gene was generated at Phenomin–Institut Clinique de la Souris (Illkirch, France; http://www.phenomin.fr/) and named VGLUT3T8I/T8I. A point mutation was introduced in exon 1 of the mouse Slc17a8 gene, leading to the ACC codon (coding for a threonine) in exchange for the ATC codon (coding for an isoleucine).
Age of mice has been modified: "All biochemical and behavioral tests were conducted with 2-8 months old littermate mice." (p.22 line 681).
Humidity conditions has been added to the Material and Methods: "Animals were housed in a temperature-controlled room (21 ± 2° C, 30-40% humidity) with ad libitum access to water and food under a 12 h light/dark cycle" (p.22 line 680).

Wild animals

No wild animals were used in the study.

Reporting on sex

See above

Field-collected samples

No field collected samples were used in the study.

Ethics oversight

In France, animal studies were performed in accordance with the European Communities Council Directive (86/809/EEC) in compliance with the Ministère de l'Agriculture et de la Forêt, Service Vétérinaire de la Santé et de la Protection Animale (authorization #13713-2018021516201278) in France.
In Canada, animal care, handling and all experiments were performed according to the guidelines of the Canadian Council on Animal Care (http://ccac.ca/en_/standards/guidelines) and approved by the Facility Animal Care Committee of the Douglas Research Center (protocols 2008-5643 and 2014-7479).
In Spain, Comitè Ètic d'Experimentació Animal-Parc de Recerca Biomèdica de Barcelona (CEEA-PRBB, Protocol Number: RML-16-0048-P1) and Generalitat de Catalunya (Protocol Number: DAAM-9687). Animal-Parc de Recerca Biomèdica de Barcelona facilities have Animal Welfare Assurance (#A5388-01, Institutional Animal Care and Use Committee approval date 05/08/2009) granted by the Office of Laboratory Animal Welfare (OLAW) of the US National Institutes of Health.

Note that full information on the approval of the study protocol must also be provided in the manuscript.

Clinical data

Policy information about clinical studies

All manuscripts should comply with the ICMJE guidelines for publication of clinical research and a completed CONSORT checklist must be included with all submissions.

Clinical trial registration

NCT01569347 and NCT00894452

Study protocol

Full trial protocols can be accessed upon request to Romain Icick (romain.icick@aphp.fr).

Data collection

- ED sample: eating disorders (ED sample) recruited by the Douglas eating disorders program (detail upon request to Pr Howard Steiger, howard.steiger@douglas.mcgill.ca).
- Substance use disorders (SUD sample) recruited in specialized SUDs outpatient clinics (N =524 genotyped) between 2008 and 2014. All patients, seeking treatment for problem cocaine use were asked to participate if they did not show exclusion criteria (note: the addiction setting sees about 150 new patients with problem cocaine use per year, so that about 50% of all patients were included).

Outcomes

- Sample #1 (ED sample): the primary outcome was the association between genetic mutation p.T8I and the type of ED diagnosed. Secondary outcomes were associations between the same mutation and (i) patients' clinical profile of the ED sample and (ii) the comorbidity profile.
- Sample #2 (SUD sample): the primary outcomes were to perform GWASs (i) on the variability of the response to methadone and (ii) on the variability of the occurrence of psychotic symptoms using the SAPS-CIP scale. For the current study, we focused on the following secondary outcomes: associations between mutations in the gene coding for VGLUT3 and (i) the diagnoses of comorbid SUDs in the sample and (ii) cocaine-induced agitation and psychotic symptoms.

Plants

Seed stocks

Report on the source of all seed stocks or other plant material used. If applicable, state the seed stock centre and catalogue number. If plant specimens were collected from the field, describe the collection location, date and sampling procedures.

Novel plant genotypes

Describe the methods by which all novel plant genotypes were produced. This includes those generated by transgenic approaches, gene editing, chemical/radiation-based mutagenesis and hybridization. For transgenic lines, describe the transformation method, the number of independent lines analyzed and the generation upon which experiments were performed. For gene-edited lines, describe the editor used, the endogenous sequence targeted for editing, the targeting guide RNA sequence (if applicable) and how the editor was applied.

Authentication

Describe any authentication procedures for each seed stock used or novel genotype generated. Describe any experiments used to assess the effect of a mutation and, where applicable, how potential secondary effects (e.g. second site T-DNA insertions, mosiacism, off-target gene editing) were examined.
